# Data-driven global ocean model resolving atmospherically forced ocean dynamics

**DOI:** 10.1126/sciadv.aed1225

**Published:** 2026-06-12

**Authors:** Jeong-Hwan Kim, Daehyun Kang, Young-Min Yang, Jae-Heung Park, Yoo-Geun Ham

**Affiliations:** ^1^ Center for Climate and Carbon Cycle Research, Korea Institute of Science and Technology, Seoul, Republic of Korea.; ^2^ Department of Environment and Energy, Jeonbuk National University, Jeonju, Republic of Korea.; ^3^ School of Earth and Environmental Sciences, Seoul National University, Seoul, Republic of Korea.; ^4^ Department of Environmental Management, Seoul National University, Seoul, Republic of Korea.

## Abstract

Artificial intelligence has advanced global weather forecasting, outperforming traditional numerical models in both accuracy and computational efficiency. Nevertheless, extending predictions beyond subseasonal timescales requires the development of deep learning (DL)–based ocean-atmosphere coupled models that can realistically simulate complex oceanic responses to atmospheric forcing. This study presents KIST-Ocean, a DL-based global three-dimensional ocean general circulation model. Comprehensive evaluations demonstrate the model’s robust ocean simulation skill and efficiency. Moreover, it reproduces ocean responses, such as Kelvin and Rossby wave propagation, and vertical motions induced by wind stress curl, demonstrating its ability to represent key atmospherically forced ocean dynamics underlying climate phenomena, including the El Niño–Southern Oscillation. These findings reinforce confidence in DL-based global weather and climate models by demonstrating their capacity to capture essential ocean-atmosphere relationships. Building on this foundation, the present study paves the way for extending DL-based modeling frameworks toward integrated Earth system simulations, thereby offering substantial potential for advancing long-range climate prediction capabilities.

## Teaser

Deep learning enables accurate ocean modeling, capturing complex oceanic responses to atmospheric forcing.

## INTRODUCTION

With the advent of the artificial intelligence (AI) era, the application of deep learning (DL) in global weather prediction has yielded remarkable achievements (*1*–*7*). These DL-based models have surpassed traditional state-of-the-art (SOTA) numerical weather prediction models in terms of both forecast skills and computational efficiency (*1*, *2*, *4*, *7*, *8*). However, several challenging issues remain, such as extending lead times beyond seasonal timescales and improving the prediction of tropical cyclone intensity and extreme climate events (e.g., cold waves, heat waves, and extreme precipitation) (*9*–*12*). Among these challenges, extending the prediction horizon is particularly substantial, as it provides societies with more time to prepare for natural disasters and delivers substantial socioeconomic value. In this context, beyond global weather prediction, it is now imperative to further investigate the application of DL in global climate prediction. To advance this objective, increased focus should be directed toward ocean modeling, given its central role in climate predictability.

The ocean, which covers the majority of the Earth’s surface, has a substantially higher heat capacity than the atmosphere, resulting in slower temperature changes. This ocean memory effect is a vital source of long-term predictability (*13*, *14*). Researchers have used the persistence of these oceanic states in the development of DL-based climate prediction models, enabling notable advancements in the multiyear forecasting of phenomena such as the El Niño–Southern Oscillation (ENSO) (*15*–*19*) and the Indian Ocean Dipole (*20*). Moreover, the interaction between the ocean and atmosphere, through which energy, momentum, and carbon fluxes are exchanged, plays a pivotal role in the Earth system (*21*). These complex dynamical and thermodynamical processes exert a substantial influence on global weather and climate, thereby underpinning large-scale climate variability mechanisms such as ENSO and the Madden–Julian Oscillation (*22*).

Recognizing this importance, traditional dynamical climate prediction systems have adopted atmosphere-ocean coupled models. Owing to the distinct characteristics of the ocean and atmosphere, these models integrate separate ocean general circulation models (OGCMs) and atmospheric general circulation models using a coupler that exchanges fluxes at the air-sea interface (*23*–*25*). This approach reflects the fundamental complexity of the coupled ocean-atmosphere system, while enabling enhanced prediction skill for long-term climate predictions.

Likewise, for DL models to successfully expand their application from weather to climate prediction, the development of an ocean-atmosphere coupled model is essential, with ocean modeling constituting a critical first step. Recently, researchers have developed several global three-dimensional (3D) ocean models based on DL (*26*–*29*). Some of these models have demonstrated improved predictive skill compared with operational ocean forecasting systems (*27*) or dynamical ocean-atmosphere coupled models (*28*). However, the adoption of nonautoregressive forecasting approaches often constrains these models, either constructing separate models for each lead time, which results in discontinuous predictions (*27*), or using a monthly temporal interval, whose temporal resolution is too coarse to capture an ocean model’s response to surface boundary forcing (*28*).

A recent attempt to implement a DL-based global ocean-atmosphere coupled model reproduced realistic Kelvin and Rossby waves in the tropical Pacific that were comparable to the corresponding reanalysis (*29*). However, as this model represents the ocean using only potential temperature, sea surface temperature (SST), and sea surface height, it lacks prognostic currents and salinity, thereby limiting its ability to resolve subsurface circulation and heat content redistribution. Moreover, the study did not conduct dedicated atmospheric forcing experiments, making it unclear whether the prescribed wind stress generated the Kelvin and Rossby waves or whether they arose from the initial ocean state.

These limitations make it challenging to assess whether DL-based ocean models can provide physically consistent responses to surface boundary conditions, raising the question of whether they can effectively simulate oceanic responses to atmospheric forcing. In this study, we address this uncertainty by developing a DL-based global 3D ocean model and evaluating its physical consistency more extensively under various atmospheric forcing experiments. To the best of our knowledge, this is the first study to verify oceanic responses driven by the atmosphere, such as tropical waves and Ekman divergence, against predictions from traditional theories.

## RESULTS

### Global ocean modeling using a U-shaped visual attention adversarial network

**Fig. 1.**
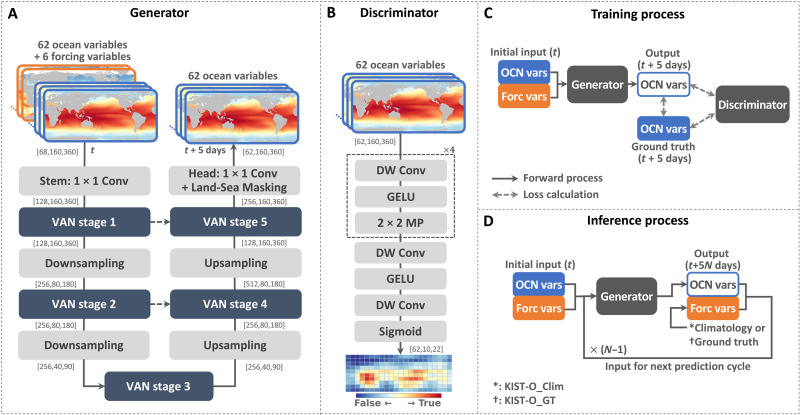
Overview of KIST-Ocean, including its training and inference processes. (**A**) Generator architecture. For details on the visual attention network (VAN) stages, down-sampling blocks, and up-sampling blocks, see fig. S12. (**B**) Discriminator architecture. DW Conv and MP denote depth-wise convolution and max pooling, respectively, while GELU indicates the Gaussian Error Linear Unit activation function. (**C**) Training process. The model was trained using a generative adversarial network (GAN) framework, in which the generator and discriminator learn in competition. The generator receives ocean and surface boundary forcing variables (denoted as OCN vars and Froc vars, respectively) and simulates ocean variables 5 days ahead. (**D**) Inference process. The figure illustrates how KIST-Ocean generates future ocean states after training is complete. Maps were generated using the Basemap Toolkit (version 1.2.0).

In this study, we propose a data-driven model based on a U-shaped visual attention adversarial network, the Korea Institute of Science and Technology’s Ocean model (KIST-Ocean), designed to simulate the global 3D ocean. We designed KIST-Ocean as a component of a coupled ocean-atmosphere model, producing only oceanic variables, similar to dynamical OGCMs (Fig. 1A). KIST-Ocean integrates 62 oceanic variables and six surface boundary conditions as inputs to simulate future oceanic states (table S1). These oceanic variables comprise two surface variables and four 3D variables with 15 vertical levels, covering the ocean subsurface from 5 to 600 m. The simulation time interval was 5 days; accordingly, both the input and output variables were averaged over 5 days.

KIST-Ocean builds on three key innovations. The first involves the integration of a visual attention network (VAN), a large-kernel attention-based convolutional architecture (*30*, *31*). VAN decomposes the convolution process into spatial and channel-wise operations, enabling the model to achieve a large receptive field with fewer parameters. Combining the U-shaped architecture with VAN further enhances parameters and computational efficiency. The U-shaped design enables multiscale feature extraction and global contextual representation learning by reducing dimensionality during down-sampling and restoring it during up-sampling. Meanwhile, skip connections prevent information loss and effectively integrate local and global information, facilitating robust performance even with relatively small datasets (*32*). This design substantially improves the model’s efficiency, enabling KIST-Ocean to simulate the global 3D ocean with 6.6 million parameters. On a single NVIDIA A100 graphics processing unit (GPU), pretraining KIST-Ocean requires approximately 33.3 hours, with an additional 2.4 hours for fine-tuning.

The second innovation is the application of partial convolution. The gridded oceanic datasets include coastal regions at the interface between ocean and land, which complicates the application of DL techniques and degrades simulation capability (*33*). Convolutional neural networks, in particular, share kernels spatially across the horizontal grid (*34*), which can lead to underestimation of the strong spatial variability in coastal regions. By incorporating partial convolution, a method that excludes masked values during the convolution process, we mitigated distortions caused by land grid cells. This approach enabled the model to more accurately capture the complex variability in coastal regions (*35*, *36*).

The third innovation involves the application of adversarial training. DL-based forecasting models that extend lead time in an autoregressive manner, by reusing outputs as inputs for the subsequent inference steps, are susceptible to unrealistic distribution drift during iterative inference. In this study, we addressed this issue by adopting a conditional generative adversarial network (GAN), thereby regularizing the output distribution of the model to align closely with that of the ground truth (*31*, *36*). Following the PatchGAN approach (*37*), we designed a discriminator to partition the image into multiple patches and evaluated each patch independently, rather than outputting a single scalar for the entire image (Fig. 1B). These methodologies are described in detail in Materials and Methods.

Consequently, KIST-Ocean was trained through adversarial training of the generator and discriminator (Fig. 1C). To ensure sufficient training data and stable optimization, we used a transfer learning strategy. First, the model was pretrained with long-term simulation data from the Community Earth System Model version 2 (CESM2) Large Ensemble (1850–2014; two ensemble members; 23,360 samples) (*38*). The model was then fine-tuned using 31 years of ocean reanalysis data (1982–2013; 2336 samples) as detailed in text S1 and table S2.

During inference, KIST-Ocean operates in an autoregressive manner, using its outputs as inputs for subsequent inference steps (Fig. 1D). By repeating this process 40 times, it generates simulations up to 200 days into the future. On a single NVIDIA A100 GPU, KIST-Ocean can produce 200-day simulations in 6 to 7 s. Because KIST-Ocean does not output surface boundary conditions, these must be prescribed as either ground truth or climatology, leading to two types of simulations: KIST-O_GT and KIST-O_Clim. Climatology serves as the time-invariant boundary condition that a coupler can provide; thus, KIST-O_GT and KIST-O_Clim represent the upper and lower bounds of KIST-Ocean’s ocean simulation capability, respectively. To evaluate the performance of KIST-Ocean, we generated KIST-O_GT and KIST-O_Clim for the period 2014–2023. The KIST-Ocean simulations were then compared with the persistence and North American Multi-Model Ensemble (NMME) dynamical seasonal prediction models.

### Performance under ground-truth surface forcing

**Fig. 2.**
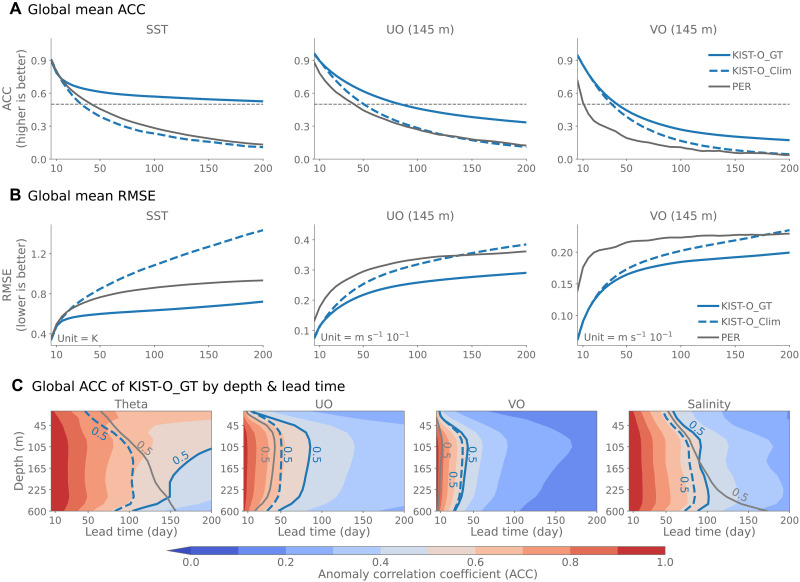
Comparative evaluation of KIST-Ocean’s global 3D ocean simulation skill against that of a persistence forecast. (**A**) Globally averaged anomaly correlation coefficient (ACC) skill for sea surface temperature (SST), zonal current (UO) at a depth of 145 m, and meridional current (VO) at a depth of 145 m (left to right), computed over 2014–2023. The solid blue and dashed blue lines represent KIST-O_GT (with ground truth prescribed as the surface boundary forcing) and KIST-O_Clim (with climatology prescribed), respectively. The solid gray line denotes the persistence forecast, and thin gray dashed lines indicate the ACC = 0.5 threshold. (**B**) Same as (A), but for globally averaged root mean square error (RMSE). (**C**) Globally averaged ACC of KIST-O_GT for all 3D ocean variables, presented as a function of depth and lead time. From left to right: potential temperature (Theta), UO, VO, and salinity. Shading represents the ACC of KIST-O_GT, while contours indicate the ACC = 0.5 threshold for KIST-O_GT (solid blue), KIST-O_Clim (dashed blue), and the persistence forecast (solid gray).

When evaluated over 200-day global ocean simulations from 2014 to 2023, KIST-Ocean demonstrated robust ocean modeling skill relative to a persistence forecast. When driven by prescribed ground-truth surface boundary forcing (i.e., KIST-O_GT), the model consistently outperformed the persistence forecast for key variables, including SST, zonal current at 145 m (UO), and meridional current at 145 m (VO), in terms of both anomaly correlation coefficient (ACC) and root mean square error (RMSE) (Fig. 2). KIST-O_GT achieved substantial ACC skill for potential temperature, UO, VO, and salinity, remaining skillful for lead times of approximately 200, 85, 40, and 100 days, respectively. Overall, among the 2480 targets (62 variables × 40 lead times), KIST-O_GT surpassed the persistence forecast for 2017 targets (81.3%) in terms of ACC and 2144 targets (86.5%) in terms of RMSE (figs. S1A and S2A). These results indicate that KIST-Ocean effectively uses surface boundary forcing information to sustain simulation skill across a broad range of oceanic variables. We note that the relative improvements over the persistence forecast are smaller at short lead times, particularly for potential temperature and salinity, which exhibit strong intrinsic oceanic memory. Conversely, the persistence forecast of ocean currents at short lead time is less skillful due to rapid transition away from initial condition driven by wind stress forcing and by mesoscale eddies.

**Fig. 3.**
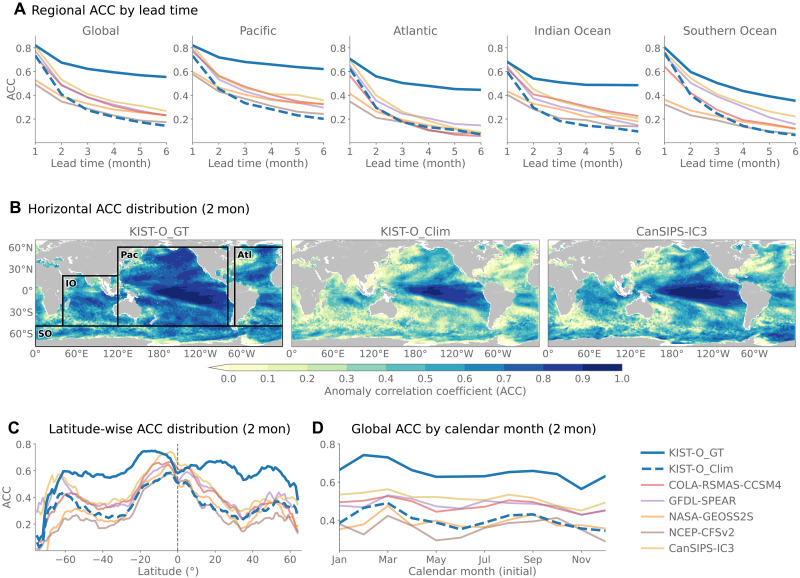
Comparative evaluation of ACC for monthly SST between KIST-Ocean and the NMME models. (**A**) Regional mean ACC as a function of lead time (months) for the globe, Pacific (120°E–80°W, 50°S–60°N), Atlantic (70°W–0°, 50°S–60°N), Indian (40°–120°E, 50°S–20°N), and Southern Ocean (0°–360°, 50°–79°S), shown from left to right. The colored lines represent KIST-O_GT (solid blue), KIST-O_Clim (dashed blue), COLA-RSMAS-CCSM4 (red), GFDL-SPEAR (purple), NASA-GEOSS2S (orange), NCEP-CFSv2 (brown), and CanSIPS-IC3 (yellow). (**B**) Horizontal distributions of 2-month lead ACC for KIST-O_GT, KIST-O_Clim, and CanSIPS-IC3, computed over 2015–2022 (left to right). (**C**) Latitudinal distribution of ACC at a 2-month lead. The *x* axis represents latitude (°), while the *y* axis represents ACC. (**D**) ACC at a 2-month lead time by initial month. The *x* axis represents the initial month (calendar month), while the *y* axis represents ACC. Maps were generated using the Basemap Toolkit (version 1.2.0).

To further assess SST skill, we compared the monthly SST simulations of KIST-O_GT with those of the NMME dynamical seasonal prediction models. KIST-O_GT outperformed the NMME predictions in SST both globally and across the major ocean basins, for lead times of up to 6 months (Fig. 3A). This result indicates that KIST-Ocean effectively learned the relationship between surface boundary conditions and SST variability when accurate surface forcing information is available.

### Performance under climatological surface forcing

When climatological surface boundary forcing was prescribed without incorporating observed forcing at the prediction time (i.e., KIST-O_Clim), SST predictions exhibited a more rapid increase in RMSE relative to both KIST-O_GT and the persistence forecast, underscoring the strong sensitivity of SST predictability to surface boundary forcing quality (Fig. 2B). The pronounced skill gap between KIST-O_GT and KIST-O_Clim highlights the model’s effective utilization of surface boundary condition information.

Despite the absence of time-varying surface forcing, KIST-O_Clim retained meaningful predictive skill in several aspects. While near-surface potential temperature exhibited shorter-lived skill relative to KIST-O_GT, subsurface ocean currents maintained comparatively longer predictability, outperforming the persistence forecast for lead times of up to approximately 120 days (Fig. 2). Specifically, among the 1200 ocean current prediction targets, KIST-O_Clim outperformed the persistence forecast for 1074 targets (89.5%) in terms of ACC and for 981 targets (81.8%) in terms of RMSE (figs. S1B and S2B). This result indicates that subsurface ocean memory provides an additional source of predictability that is partially independent of surface boundary forcing. Moreover, the model successfully captured hierarchical vertical relationships among 3D ocean variables with depth, even in the absence of explicit depth embeddings.

Under climatological surface boundary forcing, SST predictability was further assessed by comparison with the NMME dynamical seasonal prediction models. At a 1-month lead time, the SST ACC of KIST-O_Clim was comparable to that of the highest-performing NMME models, while at a 2-month lead time it remained within the overall NMME skill range (Fig. 3A). Notably, in the Atlantic Ocean, KIST-O_Clim maintained SST predictive skill comparable to NMME models for lead times of up to 6 months, despite relying solely on climatological forcing. Hence, KIST-Ocean maintained stable SST predictive skill for 1- to 2-month lead times, regardless of boundary forcing quality (Fig. 3B).

KIST-Ocean exhibited robust SST simulations across most latitudes. The influence of boundary forcing quality (i.e., the skill gap between KIST-O_GT and KIST-O_Clim) was relatively large at latitudes of approximately 20° to 50° (Fig. 3C). This skill gap decreased near the equator and Antarctica, likely associated with prevailing subsurface processes, such as those related to ENSO and the Antarctic Circumpolar Current, respectively. While the predictive skill of KIST-Ocean showed limited dependence on the seasonality of the initial conditions, higher performance tended to occur when simulations were initiated in late boreal winter, whereas simulations initialized in boreal summer or autumn exhibited relatively lower skill, a pattern also observed in the NMME models (Fig. 3D). This can be interpreted as a consequence of the deepening of the ocean mixed layer and the thermal memory effect reaching its peak in the tropical Pacific during late winter, enabling the initial conditions to more accurately capture ocean-atmosphere interactions and internal mode variability (*39*, *40*).

### Evaluating atmosphere-driven ocean dynamics

Assessing whether KIST-Ocean has adequately learned the physical relationships between surface boundary conditions and ocean variables is essential. This assessment enables us to evaluate the physical consistency of KIST-Ocean and provides insight into the feasibility of implementing a DL-based ocean-atmosphere coupled model. Accordingly, we conducted nudging experiments in which KIST-Ocean was prescribed with both idealized and historically observed surface boundary forcing to evaluate its ability to reproduce two representative physical ocean responses to atmospheric forcing: oceanic wave responses and Ekman transport responses (detailed in Materials and Methods).

In the tropical Pacific, downwelling oceanic Kelvin waves, triggered by westerly wind bursts (WWBs), are a key mechanism promoting El Niño development, as they suppress the upwelling of cold subsurface water in the eastern Pacific cold tongue (*18*, *41*). To assess whether KIST-Ocean can simulate wave responses to wind stress forcing in the tropical Pacific, we conducted nudging experiments in which westerly wind stress was prescribed within the western Pacific (130°–170°E, 10°S–10°N). The wave response was analyzed using the 20°C isotherm depth (Z20) anomaly, a reliable indicator of thermocline depth (*41*, *42*).

**Fig. 4.**
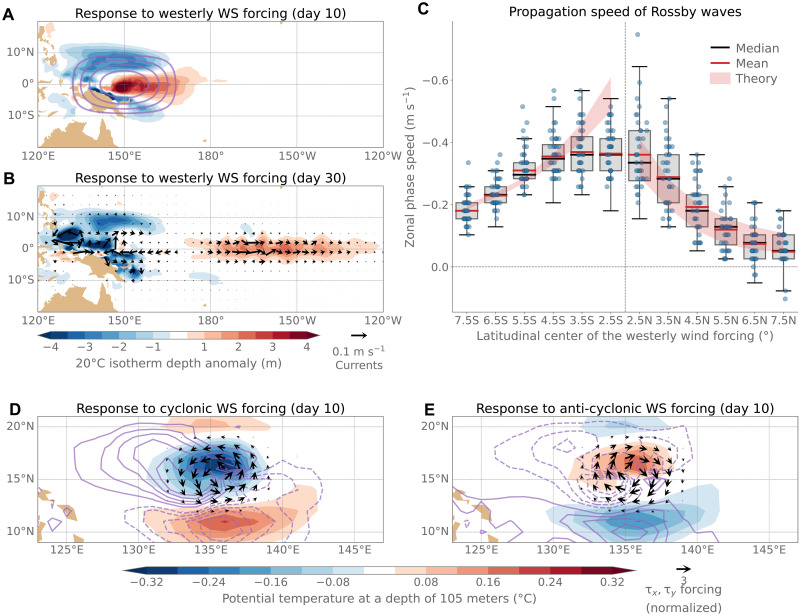
KIST-Ocean’s dynamical response to idealized wind stress forcing. (**A** and **B**) Tropical Pacific response to a bell-shaped westerly wind burst (WWB) centered at 130°–170°E, 10°S–10°N. Shading denotes the 20°C isotherm depth anomaly (m), purple contours indicate the normalized zonal wind stress forcing (unitless), and black arrows show depth-averaged (5 to 65 m) horizontal currents (m s^−1^). Snapshots are shown at lead days 10 (A) and 30 (B). The initial ocean state corresponds to 12 to 16 December 2013. (**C**) Propagation speed of Rossby waves generated by WWBs at different latitudes. Boxes represent the interquartile range; whiskers extend to 1.5 times this range; black and red lines denote the median and mean, respectively. Light-red shading indicates the theoretical long-wave phase speed derived from the first baroclinic Rossby wave approximation. The *x* axis denotes the central latitude of the WWB (°), and the *y* axis indicates the Rossby wave zonal phase speed (m s^−1^). (**D** and **E**) Western North Pacific response (131°–140°E, 11°–20°N) at lead day 10 to cyclonic (D) and anticyclonic (E) rotational wind stress forcing. Shading represents the potential temperature anomaly at 105 m (°C). Black arrows indicate the normalized surface wind stress forcing (unitless), and purple contours denote the vertical current speed inferred from mass continuity, with an interval of 0.5 (10^−6^ m s^−1^), where solid and dashed contours indicate positive and negative values, respectively. Experimental details are provided in the “Idealized wind stress forced experiments” section in Materials and Methods. Maps were generated using the Basemap Toolkit (version 1.2.0).

The nudging experiment results demonstrated that downwelling Kelvin waves were generated east of the westerly wind forcing region and propagated rapidly eastward, while upwelling Rossby waves formed to the west and propagated more slowly westward (Fig. 4, A and B). Furthermore, KIST-Ocean reproduced the reflection of upwelling Rossby waves off the Maritime Continent into upwelling Kelvin waves, which then propagated rapidly eastward along the equator, and it simulated the associated equatorial subsurface currents. It also captured the opposite-phase response to easterly wind forcing (fig. S5). Analogous zonal wind stress forcing experiments were also conducted over the Indian Ocean. The forced wave responses resulted in a pattern resembling the Indian Ocean Dipole, indicating that the modeled wave dynamics adequately resolve the horizontal scale and thermocline depth across different ocean basins (fig. S6). Overall, the simulated wave responses are consistent with the delayed oscillator theory, which emphasizes the role of equatorial wave dynamics in the development of El Niño events in the western Pacific (*41*).

To further assess KIST-Ocean’s ability to represent the key physical properties as function of latitude and thermocline depth, we conducted additional experiments prescribing westerly wind stress at varying latitudes in the eastern Pacific Ocean and analyzed the resulting changes in Rossby wave propagation speed (table S3 and fig. S7). The propagation speed of Rossby waves is expected to decrease with stronger Coriolis forces at higher latitudes, whereas it increases with deeper thermoclines (*43*).

Notably, KIST-Ocean produced physically reasonable results, showing slower Rossby wave phase speeds at higher latitudes (Fig. 4C). In addition, KIST-Ocean exhibited faster Rossby wave propagation in the Southern Hemisphere than in the Northern Hemisphere. This behavior aligns with the fact that Rossby wave phase speed is proportional to thermocline depth, which is greater in the southern tropical Pacific than in the northern tropical Pacific (fig. S7). In particular, the latitudinal variations in Rossby wave phase speeds simulated by KIST-Ocean closely matched the theoretical phase speeds estimated via long-wave approximation (detailed in Materials and Methods) (*43*). These results indicate that KIST-Ocean learned the variations in ocean wave propagation speeds as function of latitude and thermocline depth, thereby capturing the physical processes governing wave dynamics.

In the off-equatorial ocean, strong cyclonic wind stress forcing, such as that associated with tropical cyclones, induces Ekman transport, pushing surface water away from the circulation center and drawing up cooler, deeper water (commonly referred to as a “cold wake”). As a result, the center of a cyclonic circulation cools, whereas the center of an anticyclonic circulation warms, which, in turn, influences local cloud formation, precipitation, and the development of tropical cyclones (*44*). Evaluating whether a model can reproduce this mechanism provides insight into how effectively it captures the physical processes governing vertical oceanic motion and the associated temperature changes. Accordingly, we performed experiments in which both cyclonic and anticyclonic wind stress forcing were prescribed in KIST-Ocean.

Although KIST-Ocean does not explicitly formulate vertical velocity, vertical motion can be inferred from horizontal currents via the continuity equation (*45*) (see text S2). KIST-Ocean reproduced this mechanism by generating pronounced upwelling and surface cooling at the center of the cyclonic wind stress forcing region, whereas under anticyclonic forcing, the model produced downwelling accompanied by the corresponding surface warming (Fig. 4, D and E). Warm (cold) temperature anomalies developed to the north and south of the cyclonic (anticyclonic) forcing center as the accumulated (divergent) water moved away from the upwelling (downwelling) zone, inducing downwelling (upwelling) in these regions.

The ability of KIST-Ocean to reproduce the characteristic upwelling-downwelling dipole forced by surface cyclonic and anticyclonic wind stress also supports its skill in simulating larger-scale climate phenomena. An accurate representation of Ekman divergence and convergence, along with the associated vertical heat redistribution, is important for capturing the initiation and evolution of equatorial Kelvin and Rossby waves, the recharge-discharge of warm water volume, as well as the activation of the Bjerknes feedback, all core elements of the ENSO cycle (*41*). By linking surface wind stress to vertical motion and temperature anomalies, the model represents the dynamical pathway through which atmospheric perturbations modulate subsurface heat content and, ultimately, SST. Thus, the cold wake response to cyclonic forcing and the corresponding warming under anticyclonic forcing provide evidence, suggesting that KIST-Ocean has learned the key oceanic physical processes driven by atmospheric forcing that are relevant to ENSO development and decay.

To evaluate whether KIST-Ocean can reproduce atmosphere-driven oceanic physical processes not only under idealized surface boundary forcing but also under historical conditions, we examined the 2015 El Niño onset. Between March and July 2015, a sequence of WWBs over the western equatorial Pacific (140°–170°E) triggered the Bjerknes feedback and ultimately led to the strong 2015/16 El Niño event (*46*, *47*) (fig. S8). This sequence provided an ideal natural experiment for testing whether KIST-Ocean could reproduce the oceanic responses to atmospheric forcing associated with ENSO evolution. Accordingly, we conducted simulation experiments initialized on 3 May 2015, before the onset of pronounced WWBs. For this initial state, we ran two experiments: KIST-O_GT and KIST-O_Clim, driven by ground truth and climatology surface boundary conditions, respectively. These experiments enable assessment of the model’s physical consistency under historically realized surface boundary forcing.

**Fig. 5.**
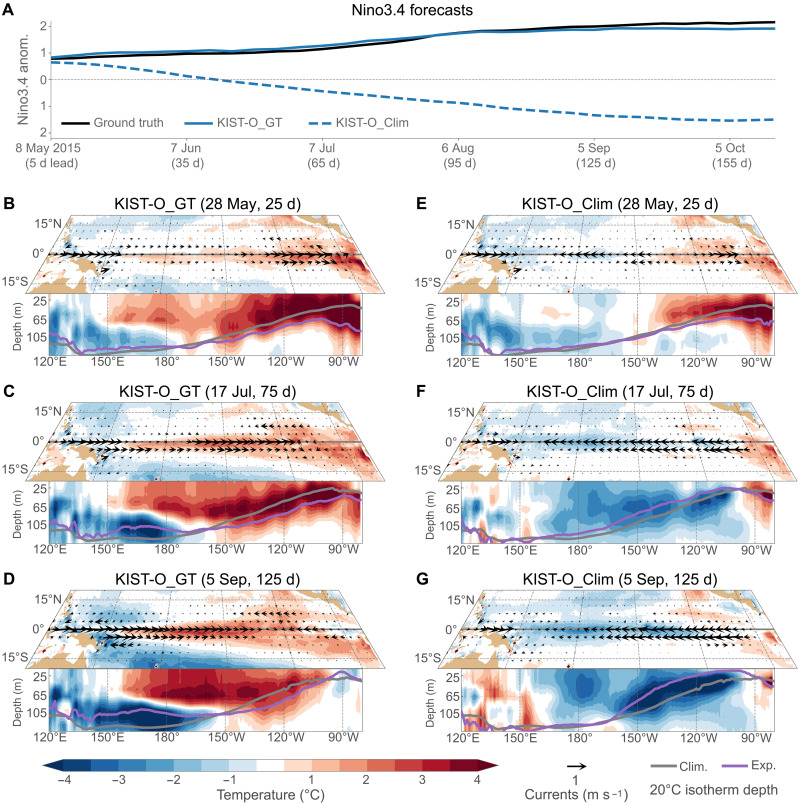
2015 Equatorial Pacific temperature evolution under ground truth versus climatological surface boundary forcing. Simulations are initiated from the ocean state on 3 May 2015. Two surface forcing configurations are compared: KIST-O_GT (ground truth forcing) and KIST-O_Clim (climatological forcing). (**A**) Time series of the Nino3.4 (defined by averaging the SST over the region 170°–120°W and 5°S–5°N) anomaly (°C) from 8 May to 19 November 2015 (lead times, 5 to 155 days). Black: GODAS (ground truth); blue solid: KIST-O_GT; blue dashed: KIST-O_Clim. (**B** to **D**) KIST-O_GT simulations at lead times of 25 days (d; 28 May 2015), 75 days (17 July 2015), and 125 days (5 September 2015). (**E** to **G**) Corresponding KIST-O_Clim simulations. For each panel, shading in the upper strip shows the SST anomaly (°C), and shading below presents the subsurface potential temperature anomaly (°C) along the equator. Black vectors denote horizontal ocean current anomalies (m s^−1^). Gray and purple contours denote the climatological 20 °C isotherm depth and the 20 °C isotherm depth in each simulation, respectively. Maps were generated with the Basemap Toolkit (version 1.2.0).

The experiments show the model’s behavior when the equatorial Pacific thermocline is deeply recharged. With realistic WWBs applied, KIST-O_GT successfully tracked the observed growth of the Nino3.4 anomaly and reproduced the development of a strong El Niño after October 2015 (Fig. 5A). The Nino3.4 index is defined by averaging the SST over the region 170°E–120°W and 5°S–5°N. In KIST-O_Clim, the absence of WWB-related westerly stress led to the development of strong easterly currents, rapidly discharging the stored heat and driving the system toward La Niña–like conditions (Fig. 5, E to G). Longitude-depth cross sections reveal that, in KIST-O_GT, convergence of warm surface waters and the core of positive subsurface temperature anomalies gradually migrated from the eastern to the central Pacific, generating a pronounced warm center in the Nino3.4 region (Fig. 5, B to D). This evolution showed strong agreement with reanalysis, with a pattern correlation coefficient of 0.78 at a lead time of 105 days (fig. S9). Without westerly forcing, KIST-O_Clim instead exhibited the rapid shoaling of the thermocline and a discharge phase typical of a transition from El Niño to La Niña. This result suggests that the surface boundary forcing, particularly the WWBs, was a major contributor to the development of 2015/16 El Niño, which is consistent with findings reported in previous studies (*46*).

Together, these experiments demonstrate that KIST-Ocean can reproduce key phenomena, such as Kelvin wave dynamics and ENSO evolution, for lead times exceeding several months when accurate surface forcing is supplied, supporting the model’s strong ocean simulation capability. The pronounced divergence between KIST-O_GT and KIST-O_Clim further indicates that the model has reasonably learned the dynamical linkage between surface wind stress, pressure-gradient forcing, and subsurface heat content, underscoring its ability to represent oceanic dynamical responses to surface forcing that are relevant to ENSO evolution.

### Ablation analysis of model components

**Fig. 6.**
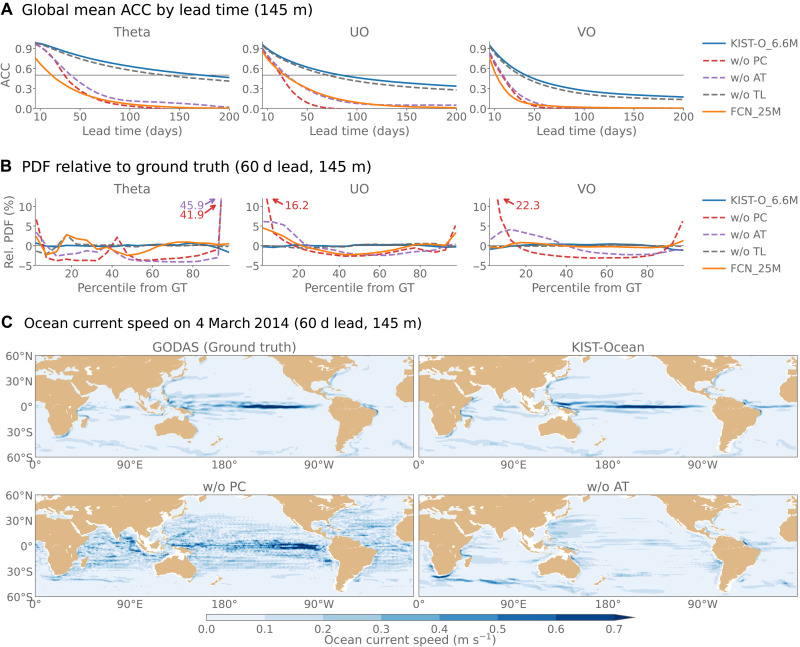
Sensitivity experiments demonstrating how key algorithms affect KIST-Ocean’s simulation performance. (**A**) Globally averaged ACC for potential temperature (Theta), zonal current (UO) at a depth of 145 m, and meridional current (VO) at a depth of 145 m, as a function of lead time over 2014–2023. KIST-O_6.6M (blue solid line) is the original model; “w/o PC” (dashed red) excludes partial convolution; “w/o AT” (dashed purple) excludes adversarial training; “w/o TL” (dashed gray) excludes transfer learning (w/o TL); “FCN_25M” (solid orange) is the FourCastNet-based model with 25 million parameters (for details, see the “FourCastNet-Based Model” section in Materials and Methods) (*42*). The *x* axis represents lead time (days), and the *y* axis represents ACC. (**B**) Relative probability density functions (PDFs) comparing 60-day simulations to ground truth; values closer to zero indicate better alignment with observations. The *x* axis represents percentiles based on the ground truth, and the *y* axis represents the relative PDF (%). (**C**) Global ocean current speed at a depth of 145 m on 4 March 2024, with a 60-day lead time. From left to right: GODAS (ground truth), KIST-O_GT, the model without partial convolution (“w/o PC”), and the model without adversarial training (“w/o AT”). Maps were generated using the Basemap Toolkit (version 1.2.0).

To enhance KIST-Ocean’s modeling capabilities, we implemented partial convolution, adversarial training, and transfer learning, and evaluated the individual contribution of each component through systematic ablation. In addition, we compared the performance of KIST-Ocean with that of a FourCastNet (*48*)–based baseline model to assess the impact of different DL algorithms on global 3D ocean modeling. Figure 6A demonstrates the positive effects of partial convolution, adversarial training, and transfer learning on simulation performance. For the key variables (potential temperature, zonal current, and meridional current), partial convolution and adversarial training had particularly strong effects; omitting either led to a rapid decline in the ACC to below 0.5 within 50 days. Transfer learning had a more modest overall impact but became increasingly beneficial, particularly for subsurface potential temperature and salinity (figs. S10 and S11). This likely reflects the lower intersample independence in deeper layers, rendering them more susceptible to sample size limitations and, consequently, more responsive to additional training data from pretrained models. For the zonal and meridional currents, the impact of applying transfer learning was not clearly evident. This result indicates that transfer learning does not always lead to substantial performance improvements, so that its application is more effective when selectively adopted depending on the degree of data sample limitations and the similarity of the tasks (*15*, *16*).

To further assess the realism of the simulated distribution, we examined the relative probability density functions of each model’s 60-day simulations with respect to the ground truth (Fig. 6B). Values closer to zero indicated closer alignment with the observations. Notably, models lacking partial convolution or adversarial training exhibited clear distributional drift, overrepresented extreme values, and underrepresented near-median values. The spatial distributions of subsurface ocean current speed (Fig. 6C) illustrate this drift. While the original KIST-Ocean closely matched the Global Ocean Data Assimilation System (GODAS; i.e., the ground truth), the version without partial convolution produced artificially strong currents across most tropical-subtropical oceans. In contrast, the version without adversarial training exhibited a pronounced blurring effect, which unrealistically weakened the equatorial currents and western boundary currents.

Although the FourCastNet-based model included 25 million parameters (approximately four times the size of KIST-Ocean’s 6.6 million), it performed notably worse in both simulation skill and distribution alignment, especially for subsurface zonal currents, for which the probabilities of near-median values decrease, while extreme values increase. These results suggest that specialized DL designs are required to effectively leverage oceanic datasets in global 3D ocean modeling.

These experiments highlight the importance of partial convolution for modeling variables with extensive missing values (commonly filled with zeros), such as ocean or land surface variables. In addition, adversarial training (or variational and diffusion methods) in autoregressive models, such as KIST-Ocean, helps mitigate distribution drift, thereby enhancing overall performance. Meanwhile, transfer learning with the CESM2-LE dataset, although marginally beneficial, was less impactful than the other two methods, suggesting that KIST-Ocean achieves robust ocean modeling skill even with a relatively limited sample size (2336 in this study). This finding highlights the efficiency of the visual attention adversarial network design for global ocean modeling and its potential to improve predictions of data-limited phenomena.

## DISCUSSION

This study presents KIST-Ocean, an efficient DL-based OGCM built on a U-shaped visual attention adversarial network framework. Although the model currently simulates only the ocean, making an exact assessment of its overall simulation skill challenging, our results demonstrate that the model achieves robust global 3D ocean simulations when provided with idealized surface boundary conditions (i.e., ground truth). Moreover, the model delivers skillful SST simulations up to a lead time of 1 to 2 months, which is comparable to that of dynamical atmosphere-ocean coupled models such as NMME, regardless of the fidelity of the surface boundary conditions. In particular, KIST-Ocean substantially outperformed persistence forecasts for 3D ocean currents without requiring realistic surface forcing, effectively capturing subsurface flows that remain challenging for SOTA dynamic models (*28*). However, because the assessments were made under prescribed boundary conditions, additional work with a fully coupled model will be required to determine whether the present DL-based ocean model can lead to improved long-term prediction skill.

Considering the extensive interactions among various Earth system components (e.g., atmosphere, ocean, sea ice, and land), it is crucial to assess whether DL-based weather and climate models can represent these interactions with physical consistency. This is critical for envisioning a DL-based Earth system model capable of long-term climate prediction and informing policy-relevant responses to climate change. In this context, we performed forcing experiments to examine whether KIST-Ocean realistically reproduces oceanic responses to atmospheric perturbations.

KIST-Ocean simulated Kelvin and Rossby waves in the tropical Pacific in response to wind forcing, capturing their eastward and westward propagations, respectively. Beyond these fundamental features, the model also replicated more nuanced dynamics, such as the varying propagation speeds of Rossby waves and the vertical oceanic motions induced by wind stress curl, both in physically coherent ways. Building on this capability, KIST-Ocean also reproduces the development of historical El Niño events with high fidelity, demonstrating its ability to robustly simulate large-scale ocean phenomena driven by atmospheric forcings. These results indicate KIST-Ocean’s ability to learn to represent the complex physical linkages between surface forcing and the ocean, while also demonstrating that DL-based approaches can capture the physical interactions among components of the Earth system. The findings further suggest that developing a DL-based atmosphere-ocean coupled model may be both feasible and promising. While the perturbed experiments yielded qualitatively reliable responses, a more rigorous quantitative assessment of the forced response was beyond the scope of this study and will be pursued in future work.

Last, the U-shaped visual attention adversarial network architecture proposed in this study proved highly effective despite the relatively limited sample sizes and model parameters. The results offer useful insights into how the land-sea mask can be incorporated into model algorithms. The finding that partial convolution and adversarial training enhance the integration of the spatially heterogeneous land-sea mask together with spatial variations in ocean depth provides valuable information for future DL-based Earth system modeling. Moreover, the sensitivity experiments examining various DL methods provide valuable guidance for future endeavors to construct DL-based coupled models, potentially enhancing the ability to model intricate ocean-atmosphere interactions.

Our comprehensive study of a DL-based model demonstrates that the model captures oceanic dynamical properties and reliable predictability in representing oceanic responses to atmospheric forcing beyond seasonal timescales. The extensive assessments of dynamical feasibility of KIST-Ocean provide strong potential for ocean-atmosphere coupling. This also indicates that the model can be applied to improve our understanding of various oceanic phenomena, while its ability to deliver global 3D ocean simulations at lower computational cost and higher speed reduces barriers for ocean research. These successful ocean simulations suggest that the model can be extended to other challenging components of the Earth system, marking a key milestone toward sustainable, data-driven climate prediction.

## MATERIALS AND METHODS

### Dataset

To enable the model to adequately learn and represent the 3D ocean state, we used potential temperature, zonal current, meridional current, and salinity as 3D prognostic variables. SST and sea ice concentration were used as 2D prognostic variables. In addition, to account for heat and momentum forcing from the atmosphere, zonal wind stress, meridional wind stress, downward longwave radiation, downward shortwave radiation, latent heat flux, and sensible heat flux were included as surface boundary forcing variables (table S1).

To ensure computational efficiency and consistency across datasets, all data were interpolated onto a regular grid (0°–360°, 79°S–80°N) with a horizontal resolution of 1°. The 3D variables were interpolated onto predefined depth levels ranging from 5 to 600 m and were represented using 15 vertical layers. The temporal resolution was set to 5-day averages (pentads). The choice of a 5-day interval is motivated by several considerations: (i) the focus of this study is on subseasonal-to-seasonal variability rather than synoptic timescales; (ii) the ocean exhibits relatively long memory due to its large heat capacity; and (iii) the 3D ocean datasets provided by the CESM2 Large Ensemble and the NCEP GODAS are natively available as 5-day means (*38*, *49*).

### Generator

KIST-Ocean was constructed using a visual attention adversarial network framework, comprising a generator and a discriminator (*30*, *31*). We designed the generator using a U-shaped VAN architecture that reduces the dimensionality of feature maps via down-sampling and subsequently restores them through up-sampling. This design facilitates the extraction of multiscale features and the learning of global contextual representations. Moreover, skip connections between the two paths help prevent information loss and effectively integrate local and global information, facilitating robust performance even with relatively small datasets (*32*).

At any given time t, the generator receives four 3D oceanic variables (each with 15 vertical levels), two 2D oceanic variables, and six 2D surface boundary forcing variables, amounting to 68 input channels (table S1). While all six oceanic variables are produced for the output, the surface boundary forcing variables are excluded from the output. Hence, the generator produces simulated oceanic fields for 5 days ahead, resulting in 62 output channels corresponding to time t+5 days. Both the input and output spanning longitudes 0°–360° and latitudes 79°S–80°N.

The generator architecture (fig. S12) begins with a stem layer that expands the number of channels via point-wise convolution (i.e., 1 × 1 convolution). Two alternating cycles of VAN stages and down-sampling blocks reduce the longitude and latitude dimensions of the feature map to one-quarter of its original size. Subsequently, three rounds of alternating VAN stages and up-sampling blocks, followed by two additional rounds, restore the dimensionality. A head layer (point-wise convolution) finally transforms the feature map into 62 output channels at t+5 days and applies land-sea masking by setting the values of land grid points to zero, ensuring that the loss calculation is unaffected by land regions. Skip connections exist between the first and fifth VAN stages and between the second and fourth VAN stages, and are implemented via concatenation before the fourth and fifth VAN stages.

Each VAN stage (fig. S12A) followed the structure proposed by Guo *et al.* (*30*), comprising batch normalization, point-wise convolution, Gaussian error linear unit (GELU) activation, large kernel attention (LKA), point-wise convolution, batch normalization, another point-wise convolution, 3 × 3 depth-wise convolution, GELU activation, another point-wise convolution, and layer normalization. Two residual connections were included within each VAN stage, and circular padding was applied to all convolutional operations to maintain longitudinal continuity. The LKA operation decomposes a K×K convolution into (2d−1)×(2d−1) depth-wise convolution, a ⌈K/d⌉×⌈K/d⌉ depth-wise dilated convolution (with dilation d), and a point-wise convolution. In this study, K=5 and d=2. As shown in fig. S12B, the LKA process is expressed as

$$\text{Attention}={\text{Conv}}_{1\times 1}\{\text{DW}-D-\text{Conv}\text{[DW}-\text{Conv}\left(V\right)\text{]\}}$$ (1)

Output=Attention⊙V (2)

Where, V is the input feature map, Attention is the attention map, and ⊙ represents the Hadamard (elementwise) product. Here, DW−Conv(·) and DW−D−Conv(·) indicate depth-wise and depth-wise dilated convolutions, respectively, while ${\text{Conv}}_{1\times 1}\left(\cdot \right)$ denotes point-wise convolution.

A down-sampling block (fig. S12C) consisted of 2 × 2 max-pooling operation followed by a point-wise convolution, halving the longitude and latitude dimensions of the input feature. Conversely, an up-sampling block (fig. S12D) used a 2 × 2 transposed convolution followed by a point-wise convolution, doubling the spatial dimensions in both longitude and latitude. Through this arrangement, the generator constructs multiscale representations of oceanic variables, ultimately producing 5-day global ocean simulations.

### Adversarial training and discriminator design

DL-based global weather prediction models commonly extend lead time by repeatedly feeding their outputs back as inputs in an autoregressive manner. However, repeated inferences can accumulate model bias, causing the predicted distribution to drift toward unrealistic values. Previous studies have addressed this issue by applying methods such as the Kullback-Leibler divergence loss or diffusion models, which help ensure that the prediction distribution remains closely aligned with reality (*3*, *5*, *50*). Similarly, the conditional GAN adopted in this study contributes to aligning the model’s simulated distribution with the actual distribution (*51*).

In this study, KIST-Ocean’s discriminator was built on the basis of the PatchGAN approach proposed by Isola *et al.* (*37*). Unlike a traditional GAN discriminator, which outputs a single scalar to determine whether an entire image is real or fake, PatchGAN divides the image into multiple patches and evaluates each patch independently. This approach enables the discriminator to effectively capture high-frequency details and local features, thereby enhancing the realism of image-to-image translation.

As illustrated in Fig. 1D, the KIST-Ocean discriminator receives as inputs the generator’s simulated fields at t+5-day and the corresponding ground-truth fields at t+5-day. It then processes these inputs through six 3 × 3 depth-wise convolution layers and four 2 × 2 max-pooling layers, applying GELU activations in all but the final convolution layer, which uses a sigmoid activation. This yields an output tensor with dimensions of 23 by 10 by 62, where each value ranges from 0 to 1, corresponding to the probability that each patch is real. Notably, the discriminator classifies real or fake patches at the 16° by 16° patch level, thereby enhancing local distribution alignment and effectively capturing fine-scale features in the generated oceanic fields.

### Training processes

In this study, we adopted a transfer learning approach to address the limited availability of observational (reanalysis) samples for training. Transfer learning, which reuses knowledge from a preexisting model to accelerate adaptation to new tasks, is particularly effective when the number of training samples is restricted (*52*). It has also demonstrated success in various climate prediction applications, including ENSO forecasts (*15*). We first pretrained KIST-Ocean using two ensemble members from the CESM2-LE dataset, each covering the period of 1850–2014 (table S2). Specifically, we used the entire 1850–2014 dataset from the first ensemble member (1301.012), together with the 1850–2004 segment of the second ensemble member (1301.013), to create our training dataset. The remaining 2005–2014 portion of the second ensemble was reserved for validation. This approach ensured that the model was exposed to a sufficiently diverse historical simulation range while preserving an independent dataset for evaluating performance during the pretraining phase.

Following pretraining, the model was fine-tuned using 2336 reanalysis samples from 2014 to 2013 (inclusive), without an additional validation step. During pretraining, the model was trained for 100 epochs, and the version yielding the minimum objective function on the validation dataset was selected as the final pretrained model. In the subsequent fine-tuning stage, the model was trained for 50 epochs, and the final version of the epoch was adopted for subsequent analysis. In this study, KIST-Ocean was pretrained and fine-tuned using a single NVIDIA A100 GPU, requiring approximately 33.3 hours for pretraining and 2.4 hours for fine-tuning, thereby underscoring the efficiency of this methodology.

We used PyTorch’s “ReduceLROnPlateau” for learning rate scheduling, decreasing the learning rate whenever improvements in the objective function plateaued. In the pretraining phase, the initial learning rate was set to 0.001, whereas it was set to 0.0001 during fine-tuning. Both phases used the AdamW optimizer (*53*) with a weight decay of 0.1, a batch size of 20, and no dropout or droppath techniques.

### Inference process

KIST-Ocean uses an autoregressive approach for inference, wherein the model’s output is used as the input for the subsequent simulation step (Fig. 1B), a strategy widely adopted in DL-based global prediction models (*1*–*4*, *7*, *28*, *54*). The primary objective of this study is to evaluate the model’s response to surface boundary forcing on subseasonal-to-seasonal timescales. For this reason, simulations were conducted up to 200 days, which is sufficient to capture the relevant dynamical adjustment processes of interest. In practice, this autoregressive procedure was repeated 40 times to generate simulation results from 5 to 200 days after the initial state. All simulations were conducted using a single deterministic realization, without ensemble averaging. Instead, to reduce the uncertainty in skill assessment arising from the use of a single realization, we evaluated simulation skill over a relatively long verification period spanning 10 years (2014–2023).

As KIST-Ocean does not internally produce surface boundary conditions, these conditions were externally prescribed either as ground truth or as climatology, resulting in two types of simulations (hereafter referred to as KIST-O_GT and KIST-O_Clim, respectively). Climatological forcing can be regarded as an uninformative case obtainable from a coupler. Accordingly, KIST-O_GT and KIST-O_Clim effectively represent the upper and lower bounds of the simulation skill achievable by future DL-based ocean-atmosphere coupled models, respectively.

### Visual attention network

The VAN uses a self-attention mechanism to capture long-range dependencies and utilizes large-kernel convolutions to establish relationships and produce attention maps (*30*). Although large kernels generally require substantial computational resources and a high number of parameters, the VAN mitigates these limitations by decomposing the convolution process through LKA, thereby enabling the use of larger kernels with relatively few parameters. Consequently, VAN-equipped models have a broader receptive field, effectively capturing both global and local relationships. By incorporating VAN, KIST-Ocean achieved a relatively large 9 by 9 receptive field with only 6.6 million parameters. Notably, in VAN stage 3, the receptive field extends to 36° by 36°, providing sufficient spatial coverage for resolving phenomena occurring on timescales of less than 1 week.

### Partial convolution

Partial convolution is used to handle masked or invalid grid points in convolutional neural networks, originally introduced in the context of image inpainting. In ordinary convolution operations, masked regions, typically filled with zeros, are treated as valid inputs. This assumption is problematic when zero values do not represent meaningful physical states, as the convolutional kernel then learns spurious patterns influenced by artificial boundaries (fig. S13A).

Partial convolution addresses this issue by restricting the convolution operation to valid grid points only and renormalizing the output according to the number of valid inputs within the convolution kernel (fig. S13B). As a result, the contribution of missing or invalid grid points is explicitly excluded, allowing the learned filters to better reflect physically meaningful spatial correlations. Owing to these advantages, partial convolution has been successfully applied not only in image inpainting but also in climate data reconstruction and the data assimilation of 3D global ocean temperature (*35*, *36*, *55*).

Formally, given an input feature map V and a binary mask M, where valid grid points are indicated by $m_{i}=1$ and masked (invalid) grid points by $m_{i}=0$, the partial convolution operator PConv(V,M) is defined as

$$\text{PConv}\left(V,M\right)=\frac{\sum_{i\in \Omega }w_{i}v_{i}\cdot m_{i}}{\sum_{i\in \Omega }m_{i}+\varepsilon }$$ (3)

Where, Ω represents the convolution kernel region, and $w_{i}$ denotes the convolution filter weights. The numerator aggregates contributions solely from valid grid points, while the denominator normalizes the output by the effective number of valid inputs. The small constant ε is introduced to ensure numerical stability when all grid points within the kernel are masked.

In the context of oceanic variables, land grid cells constitute permanently invalid regions that do not carry physically meaningful ocean information. Treating these land grid cells as ordinary zeros in standard convolution would introduce artificial discontinuities along coastlines and distort the learning of spatial filters. To mitigate this issue, all convolution operations within the generator, except for point-wise (1 × 1) convolutions, which do not aggregate spatial neighbors, are replaced with partial convolutions. This design choice ensures that convolutional kernels are trained exclusively on physically valid ocean grid points, leading to more robust and physically consistent feature representations.

### Objective functions

In this study, the objective functions $L_{G}$ and $L_{D}$ for generator G and discriminator D, which constitute KIST-Ocean, were defined as follows

$$L_{G}=-\alpha \;\text{log}\;D\left[G\left(X\right)\right]+\beta L_{1}$$ (4)

$$L_{D}=-\left\{\text{log}\;D\left(Y\right)+\text{log}[1-DG\left(X\right)]\right\}$$ (5)

Where X and Y represent the input and ground truth, respectively, and are tensors with dimensions $N_{\text{lon}}\times N_{\text{lat}}\times N_{\text{ch}}$. $N_{\text{lon}}$, $N_{\text{lat}}$, and $N_{\text{ch}}$ denote the number of longitude and latitude grid points and the number of channels, respectively. The generator’s output G(·) and the discriminator’s output D(·) are tensors with dimensions $N_{\text{lon}}\times N_{\text{lat}}\times N_{\text{ch}}$ and $\left\lceil \frac{N_{\text{lon}}}{16}\right\rceil \times \left\lceil \frac{N_{\text{lat}}}{16}\right\rceil \times N_{\text{ch}}$, respectively.

In $L_{G}$, the balance between the adversarial loss term (−α log D[G(X)]) and the reconstruction loss term ($\beta L_{1}$) is controlled by the regularization parameters α and β, respectively. We set α and β to 0.4 and 0.6, respectively. That is, $L_{G}$ decreases as the discriminator classifies the generator’s output closer to one (i.e., the discriminator perceives the generator’s output as more realistic) and as the error between the generator’s output and the ground truth decreases. In contrast, $L_{D}$ decreases as the discriminator classifies the ground truth closer to one and the generator’s output closer to zero.

The reconstruction loss $L_{1}$ is defined as follows

$$L_{1}=\frac{1}{N_{\text{lon}}N_{\text{lat}}N_{\text{ch}}}\sum_{i=1}^{N_{\text{lon}}}\sum_{j=1}^{N_{\text{lat}}}\sum_{k=1}^{N_{\text{ch}}}A\left(\emptyset_{j}\right)|G{\left(X\right)}_{i,j,k}-Y_{i,j,k}|$$ (6)

Where $A\left({\emptyset \emptyset }_{j}\right)$ represents the latitude weight at latitude ${\emptyset \emptyset }_{j}$ and is defined as $N_{\text{lat}}\frac{\text{cos}{\emptyset \emptyset }_{j}}{\sum_{j}^{N_{\text{lat}}}\text{cos}{\emptyset \emptyset }_{j}}$.

### Idealized wind stress forced experiments

In this study, we conducted three types of wind stress forcing experiments to evaluate, from multiple perspectives, whether DL-based KIST-Ocean can simulate physically consistent responses to atmospheric surface boundary forcing.

#### Westerly and easterly wind bursts

In the tropical Pacific, WWBs are a major trigger for El Niño development. In the Indian Ocean, it can also partially facilitate the development of a negative Indian Ocean Dipole. East of WWBs, downwelling Kelvin waves form and rapidly travel eastward along the equator, leading to thermocline deepening and SST warming near the eastern boundary of the basin. Concurrently, upwelling Rossby waves form to the west and propagate westward. Upon reaching the western boundary of the basin, some of these waves reflect and transform into upwelling Kelvin waves, which then travel eastward along the equator. As these reflected waves shoal the thermocline and cool the sea surface, they can weaken El Niño. This reflective mechanism is pivotal for the transition from El Niño to La Niña and constitutes a core aspect of the “delayed oscillator” theory of ENSO.

In this study, we conducted WWB and easterly wind burst (EWB) experiments by applying zonal wind stress anomalies in the western tropical Pacific (130°–170°E, 10°S–10°N) and tropical Indian Ocean (70°–90°E, 5°S–5°N) to verify whether KIST-Ocean reproduces the generation and propagation of Kelvin and Rossby waves in a manner consistent with that of the delayed oscillator theory. Specifically, we prescribed an idealized zonal wind stress forcing ${\text{frc}}_{i,j}$ following a Gaussian distribution

$$\begin{array}{c} {\text{frc}}_{i,j}=\text{γsin}\left[\frac{\pi \left(i+0.5\right)}{N_{\text{lon}}}\right]\text{sin}\left[\frac{\pi \left(j+0.5\right)}{N_{\text{lat}}}\right],\\ i\in \left\{0,1,\cdots ,N_{\text{lon}}\right\},j\in \left\{0,1,\cdots ,N_{\text{lat}}\right\} \end{array}$$ (7)

Where $N_{\text{lat}}$ and $N_{\text{lon}}$ denote the numbers of grid points in the zonal and meridional directions of the forcing region, respectively. The scale parameter γ (amplitude) was set to either 1 or −1, defining a WWB run (γ=1) and an EWB run (γ=−1). The initial ocean state was taken from 14 December 2013 (averaged over 14 to 16 December), and climatological surface boundary conditions were prescribed. In the forcing region, the WWB and EWB runs added ${\text{frc}}_{i,j}$ to the climatological zonal wind, but only during the first inference step, such that the subsequent wave responses could be traced to the imposed perturbation.

A control run using the same climatological boundary conditions, but without any additional perturbations, was performed for comparison. We examined 20°C isotherm depth anomalies to investigate the generation and propagation of Kelvin and Rossby waves. Specifically, anomalies were calculated by subtracting the control run from the WWB and EWB runs, thereby isolating the wave responses triggered by WWBs and EWBs within KIST-Ocean.

#### Rossby wave propagation experiments

In the Pacific Ocean, oceanic Rossby waves generated by wind stress perturbations exhibit a phase speed inversely proportional to latitude and proportional to thermocline depth (*56*). We conducted WWB experiments at various latitudes in the tropical Pacific to evaluate whether KIST-Ocean could capture such higher-level physical relationships. Specifically, the longitudinal range for the forcing region was fixed at 160°–140°W, while the latitude bands were incrementally shifted from 0°–5°S to 5°–10°S in the Southern Hemisphere and from 0°–5°N to 5°–10°N in the Northern Hemisphere, resulting in 12 latitude bands ($N_{\text{lon}}=21,N_{\text{lat}}=6$; table S3 and fig. S7).

We used 36 initial ocean conditions drawn from the central pentad of each month in 2014, 2015, and 2016 (e.g., 13 January, 17 February, and 14 March) for a total of 432 WWB perturbation experiments (12 latitudes × 36 initial conditions). As in previous experiments, WWB forcing was applied only during the first inference step. The control run used the same 36 initial conditions and prescribed climatological surface boundary conditions but did not include any additional perturbations. By comparing the WWB runs with the control runs, we examined the ability of KIST-Ocean to simulate Rossby wave propagation at different latitudes, thereby testing whether it can capture the essential physics of latitude-dependent wave speed.

#### Rotational wind stress forcing

Strong cyclonic wind stress forcing, such as that induced by tropical cyclones, can generate Ekman transport, driving surface water outward from the center and drawing up colder, deeper water (upwelling). Consequently, the center of the cyclonic circulation cools, whereas that of the anticyclonic circulation warms. To investigate whether KIST-Ocean can reproduce the associated 3D oceanic responses in a manner consistent with real-world physics, we conducted experiments applying both cyclonic and anticyclonic wind stress forcing.

In this study, idealized rotational wind stress perturbations were defined using Gaussian functions. For a 2D grid system, let $i_{0}=\frac{N_{\text{lon}}+1}{2}$ and $j_{0}=\frac{N_{\text{lat}}+1}{2}$ denote the zonal and meridional centers of the forcing region, respectively, and let $r_{i,j}=\sqrt{{\left(i-i_{0}\right)}^{2}+{\left(j-j_{0}\right)}^{2}}$ represent the distance of each grid point (i, j) from the forcing center. The zonal $u_{i,j}$ and meridional $v_{i,j}$ components of the rotational wind stress perturbation are defined as follows

$$u_{i,j}=\gamma \left(-\frac{j-j_{0}}{r_{i,j}}\right)\text{exp}\left(-\frac{{r_{i,j}}^{2}}{2\lambda^{2}}\right),v_{i,j}=\gamma \left(-\frac{i-i_{0}}{r_{i,j}}\right)\text{exp}\left(-\frac{{r_{i,j}}^{2}}{2\lambda^{2}}\right)$$ (8)

Where the scale parameter γ determines the circulation type: γ > 0 corresponds to cyclonic circulation, while γ < 0 indicates anticyclonic circulation. The Gaussian spread λ controls how rapidly (or gradually) the wind stress forcing amplitude decays from the center; in this experiment, λ=1.

The forcing region was set to 131°–140°E, 11°–20°N, a typical location for tropical cyclone formation, resulting in $N_{\text{lon}}=10\;\text{and}\;N_{\text{lat}}=10$. The initial oceanic conditions were taken from 17 June 2015, and the climatological surface boundary conditions were prescribed. In the cyclone and anticyclone runs, the zonal wind component $u_{i,j}$ was added to the climatological zonal wind, and the meridional component $v_{i,j}$ was added to the climatological meridional wind at every inference step, producing persistent cyclonic (γ=1) or anticyclonic (γ=−1) wind stress perturbations. In contrast, the control run employed identical initial conditions and surface boundary climatology but no perturbations.

To assess the temperature response to rotational wind stress forcing, potential temperature anomalies (at a depth of 105 m) in the cyclone and anticyclone runs were calculated by subtracting the control run. The spatial patterns of these anomalies indicate how continuous cyclonic or anticyclonic wind stress forcing alters the vertical structure and distribution of oceanic temperature in KIST-Ocean.

#### KIST-Ocean Rossby wave propagation speed

In this study, we calculated the propagation speed of Rossby waves (*43*) using the following three-step procedure:

Step 1: Take the latitudinal mean of the 20°C isotherm depth anomaly, $Z20_{i,j,t}$ (unit, m), for longitude i (unit, °), latitude j (unit, °), and lead time t (unit, days) over the Rossby wave detection zone (see table S3) and apply 1D Gaussian filtering. By expressing this mathematically

$${\left[Z20\right]}_{i,t}=\left(\frac{1}{\phi_{2}-\phi_{1}}\sum_{j=\phi_{1}}^{\phi_{2}}Z20_{i,j,t}\right)*\frac{1}{\sqrt{2\pi }\sigma }\text{exp}\left(-\frac{i^{2}}{2\sigma^{2}}\right)$$ (9)

Where $\phi_{1}$ and $\phi_{2}$ represent the latitudes of the equatorward and poleward edges of the Rossby wave detection zone, respectively (unit, °). We defined the Rossby wave detection zone as the region extending from 1.5° ($\phi_{1}$) to 5.5° ($\phi_{2}$) poleward of the central latitude of the wind stress forcing (table S3). The symbol * denotes the zonal convolution operator (i.e., 1D convolution), while σ is a parameter that controls the intensity of the Gaussian blur (here, σ = 9).

Step 2: Find the longitude $i_{t}^{*}$, at which ${\left[Z20\right]}_{i,t}$ reaches its minimum (unit, °)

$$i_{t}^{*}=\text{arg}\underset{i}{\text{min}}\left({\left[Z20\right]}_{i,t}\right)$$ (10)

Step 3: Last, the Rossby wave propagation speed $C_{Z}$ (unit, $m\;s^{-1}$) was calculated as follows

$$C_{Z}=\frac{\text{Rad}\left(i_{t=55}^{*}-i_{t=5}^{*}\right)R}{50\times 86,400}$$ (11)

Where R represents the Earth’s radius, defined in this study as 6,378,000 m. The denominator 50×86,400 converts the time unit from 50 days to seconds.

#### Rossby wave theoretical phase speed

In addition, to determine whether KIST-Ocean produces realistic Rossby wave propagation speeds, we assumed a simplified first baroclinic Rossby wave under latitude and thermocline depth conditions and estimated its phase speed using long-wave approximation. In an actual ocean, local and temporal deviations (e.g., background currents, nonlinear mixing, and spatial and temporal wind stress distributions) can introduce differences between theory and observations (*57*). Assuming sufficiently large wavelengths, the phase speed $C_{\text{Phase}}$ of the first baroclinic Rossby wave can be approximated by (*43*)

$$C_{\text{Phase}}=-\beta \frac{C_{o}^{2}}{f^{2}}$$ (12)

Where the negative sign indicates westward propagation, $C_{0}=\sqrt{g^{\prime }H}$ is the first internal gravity wave speed, f=2Ωcos∅ ∅ is the Coriolis parameter at latitude ∅ ∅, and $\beta =\frac{\partial f}{\partial y}\approx \frac{2\Omega \text{cos}\emptyset \emptyset }{R_{E}}$ is the β term. Here, $\Omega \approx 0.03\;m\;s^{2}$ is Earth’s angular rotation speed, $R_{E}\approx 6.37\times 10^{6}\;m$ is Earth’s radius, $g^{\prime }\approx 0.03\;m\;s^{2}$ is the reduced gravity, and H is the thermocline depth. Thus, as latitude increases, the phase speed decreases, and as the thermocline deepens, the phase speed increases.

### FourCastNet-based model

We used FourCastNet to investigate the sensitivity of global 3D ocean simulations to different DL algorithms. FourCastNet is a global, data-driven weather forecasting model based on the adaptive Fourier neural operator (AFNO) (*58*). It was trained on the ERA5 reanalysis dataset (1979–2018, 6-hour intervals, 0.25° resolution, and 20 atmospheric variables) to generate predictions up to 10 days in advance (*48*). In this study, we configured FourCastNet with a patch size of 4 × 4, 500D patches and position embeddings, and five AFNO blocks, resulting in 25 million parameters, nearly four times the parameter count of KIST-Ocean. Training followed the same two-phase approach used for KIST-Ocean: a pretraining phase using the CESM2-LE dataset and a fine-tuning phase using reanalysis data. During pretraining, the learning rate was set to 0.001, whereas for fine-tuning, it was set to 0.0001. Both phases used the FuseAdam optimizer for model training.

### Evaluation metrics

To assess global 3D ocean modeling performance, this study used the RMSE and ACC. For all metrics, the global average was calculated using the latitude-based weighting function $A\left({\emptyset \emptyset }_{j}\right)$ defined in the “Objective functions” section. Accordingly, the global mean RMSE between the ground truth $Y_{t,i,j,k}$ and simulation ${\hat{Y}}_{t,i,j,k,l}$ for channel k and lead time l is given by

$$\begin{array}{c} {\text{RMSE}}_{k,l}=\\ \sqrt{\frac{1}{N_{\text{time}}N_{\text{lon}}N_{\text{lat}}}\sum_{t=1}^{N_{\text{time}}}\sum_{i=1}^{N_{\text{lon}}}\sum_{j=1}^{N_{\text{lat}}}A\left(\emptyset_{j}\right){\left[\left({\hat{Y}}_{t,i,j,k,l}-\bar{{\hat{Y}}_{t,i,j,k,l}}\right)-\left(Y_{t,i,j,k}-\bar{Y_{t,i,j,k}}\right)\right]}^{2}} \end{array}$$ (13)

Where $N_{\text{time}}$, $N_{\text{lon}}$, and $N_{\text{lat}}$ denote the number of time samples, longitudes, and latitudes, respectively, and $\bar{\left(\cdot \right)}$ indicates the temporal climatology (from the first to the 73rd pentad).

Because Pearson’s correlation coefficient can be underestimated when directly averaged over an asymmetrically distributed sample (*59*), simply averaging grid-wise ACC values globally may introduce bias. To mitigate this issue, a Fisher’s Z-transform was first applied to each grid point’s ACC; the transformed values were then averaged, and the result was lastly inverse transformed.

First, the ACC at each grid point (i, j) for channel k and lead time l is defined as

$${\text{ACC}}_{i,j,k,l}=\frac{\sum_{t=1}^{N_{\text{time}}}\left({\hat{Y}}_{t,i,j,k,l}-\bar{{\hat{Y}}_{t,i,j,k,l}}\right)\left(Y_{t,i,j,k}-\bar{Y_{t,i,j,k}}\right)}{\sqrt{\sum_{t=1}^{N_{\text{time}}}{\left({\hat{Y}}_{t,i,j,k,l}-\bar{{\hat{Y}}_{t,i,j,k,l}}\right)}^{2}\sum_{t=1}^{N_{\text{time}}}{\left(Y_{t,i,j,k}-\bar{Y_{t,i,j,k}}\right)}^{2}}}$$ (14)

The global mean ACC (${\text{ACC}}_{k,l}$) was then computed by applying the Fisher’s Z-transform to each ${\text{ACC}}_{i,j,k,l}$, taking the average of these transformed values, and subsequently applying the inverse Z-transform

$${\text{ACC}}_{k,l}=\text{tanh}\left[\frac{1}{N_{\text{lon}}N_{\text{lat}}}\sum_{i=1}^{N_{\text{lon}}}\sum_{j=1}^{N_{\text{lat}}}A\left(\emptyset_{j}\right)\text{arctanh}\left({\text{ACC}}_{i,j,k,l}\right)\right]$$ (15)

Where tanh and arctanh denote the hyperbolic tangent and inverse hyperbolic tangent functions, respectively.

## Supplementary Materials

### This PDF file includes:

Supplementary Text Figs. S1 to S14 Tables S1 to S4 References
