# Supplementary material for: Data-driven global ocean model resolving atmospherically forced ocean dynamics
Source: Sci Adv. 2026 Jun 12;12(24):eaed1225. doi: 10.1126/sciadv.aed1225 (PMC13262615; doi:10.1126/sciadv.aed1225)
Supplement: Supplementary file 1 — Supplementary Text Figs. S1 to S14 Tables S1 to S4 References [file sciadv.aed1225_sm.pdf]

Science Advances
AAAS

Supplementary Materials for

Data-driven global ocean model resolving atmospherically forced ocean dynamics

Jeong-Hwan Kim *et al.*

Corresponding author: Jeong-Hwan Kim, jeonghwan@kist.re.kr; Daehyun Kang, dkang@kist.re.kr

Sci. Adv. **12**, eaed1225 (2026)
DOI: 10.1126/sciadv.aed1225

This PDF file includes:

Supplementary Text
Figs. S1 to S14
Tables S1 to S4
References

Supplementary Text

Text S1. Datasets

Text S1.1. CESM2 Large-Ensemble Simulations

For pretraining of KIST-Ocean, we utilized two ensemble members (1301.012 and 1301.013) from the historical simulations (1850–2014) of the CESM2 Large Ensemble Community Project (CESM2-LE) (38). Model outputs were averaged over five-day intervals (pentads) and interpolated horizontally onto a regular 1°×1° grid spanning 0°–360° longitude and 79°S–80°N latitude, and vertically onto 15 levels (depths of 5, 25, 45, 65, 85, 105, 125, 145, 165, 185, 205, 225, 265, 365, and 600 m) to facilitate transfer learning. This procedure yielded a total of 24,090 samples, of which 23,360 (320 years × 73 pentads) were used for training, and the remaining 730 (10 years × 73 pentads) were reserved for validation. Each variable was standardized using the z-score method, without removing the climatological mean (i.e., seasonal cycles).

Text S1.2. Reanalysis dataset

To fine-tune KIST-Ocean and assess its global 3D ocean performance, we employed several reanalysis datasets. Three-dimensional oceanic variables (potential temperature, zonal and meridional currents, and salinity), together with surface wind stress, were obtained from the National Centers for Environmental Prediction (NCEP) Global Ocean Data Assimilation System (GODAS) (49), which is natively provided as five-day averaged fields. Two-dimensional oceanic variables (sea surface temperature (SST) and sea ice concentration) were derived from the National Oceanic and Atmospheric Administration Optimum Interpolation Sea Surface Temperature (OISST) version 2 (60). Surface energy flux variables (downward shortwave and longwave radiation, sensible and latent heat fluxes) were sourced from the European Centre for Medium-Range Weather Forecasts Reanalysis version 5 (ERA5) (61) for the period 1982–2023.

For consistency, these data were interpolated onto the same horizontal 1°×1° grid and vertical levels utilized during pretraining. From the resulting total of 3,066 samples, 2,336 (covering the period 1982–2013) were allocated for fine-tuning, while the remaining 730 samples (covering 2014–2023) were reserved for evaluation. All variables were standardized using the z-score method without removing the climatological mean.

Text S1.3. The North American Multi-Model Ensemble (NMME) Forecasts

To evaluate the *SST* simulation skill of KIST-Ocean, we utilized global *SST* forecasts from the NMME project provided by the International Research Institute for Climate and Society (IRI) (39), which are available as monthly mean predictions. Specifically, we selected the COLA-RSMAS-CCSM4, GFDL-SPEAR, NASA-GEOSS2S, NCEP-CFSv2, and CanSIPS-IC3 models for the verification period spanning 2014–2023 (Table S4). Each model, configured as a fully

coupled atmosphere–ocean general circulation model, contributed 9–12 ensemble members. CanSIPS-IC4 was excluded due to incomplete data availability. Monthly forecast anomalies were computed by removing the climatological mean computed over 2014–2023 separately at each forecast lead time.

Text S2. Estimation of vertical velocity

Because KIST-Ocean does not explicitly produce ocean vertical velocity, $\boldsymbol{w}$, we infer it diagnostically from the modelled horizontal velocities, $\boldsymbol{u}$ (zonal) and $\boldsymbol{v}$ (meridional), by integrating the three-dimensional continuity equation,

$$\frac{\partial \boldsymbol{u}}{\partial x} + \frac{\partial \boldsymbol{v}}{\partial y} + \frac{\partial \boldsymbol{w}}{\partial z} = 0 \qquad \text{(S1)}$$

Horizontal derivatives $\frac{\partial \boldsymbol{u}}{\partial x}$ and $\frac{\partial \boldsymbol{v}}{\partial y}$ are evaluated with centered finite differences on the model grid. Integrating Eq. (S1) vertically from the surface ($z = 0$) to an arbitrary depth z and imposing a rigid-lid upper boundary condition $w(z = 0) = 0$, yields

$$\boldsymbol{w}(z) = -\int_0^z \left(\frac{\partial \boldsymbol{u}}{\partial x} + \frac{\partial \boldsymbol{v}}{\partial y}\right) dz' \qquad \text{(S2)}$$

Equation (S2) provides a consistent estimate of vertical velocity throughout the water column based solely on the horizontal velocity fields produced by KIST-Ocean (45).

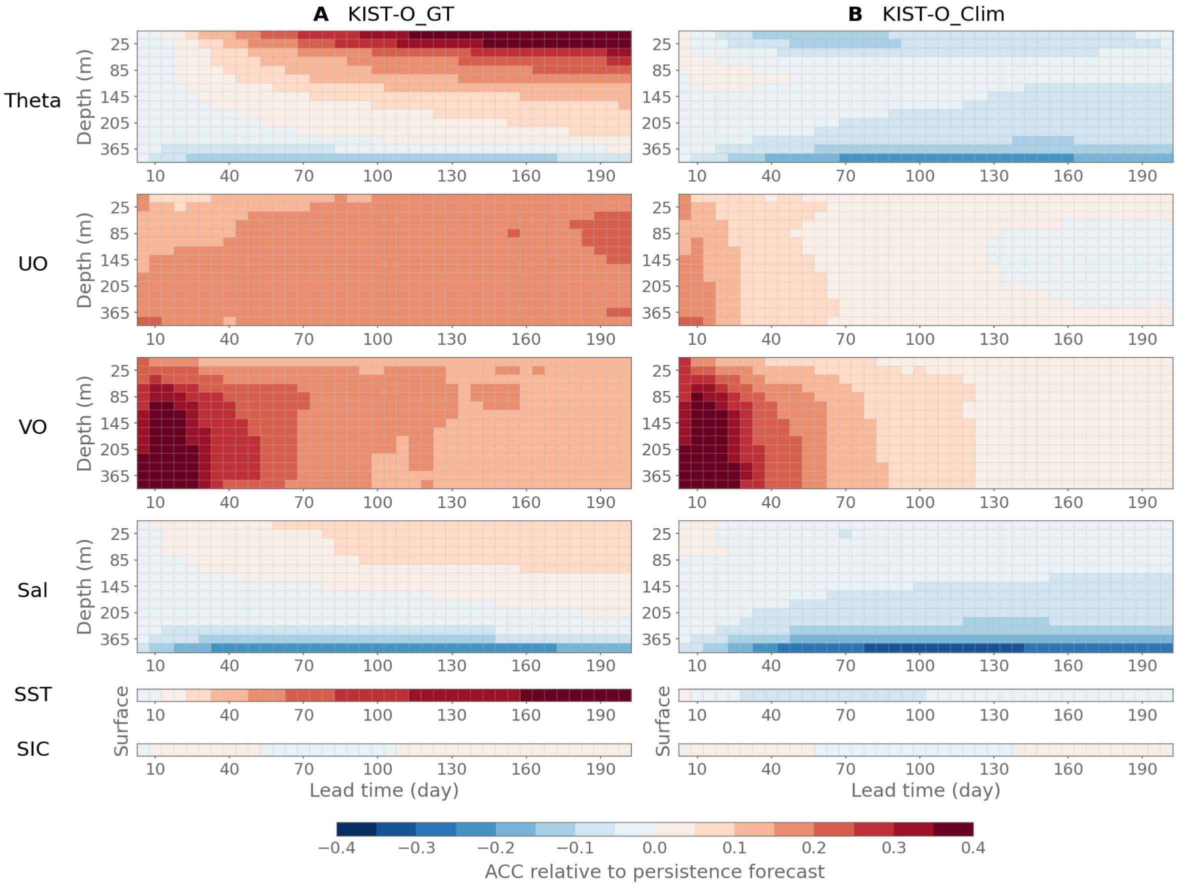

Fig. S1. Scorecard of relative anomaly correlation coefficient (ACC), computed over 2014–2023. Here, "relative ACC" is defined as the difference between the ACC of each KIST-Ocean inference setup and that of the persistence forecast. The left column displays the relative ACC for KIST-O_GT (with the ground truth prescribed as the surface boundary forcing), whereas the right column shows that for KIST-O_Clim (with the climatology prescribed). From top to bottom, each row presents the relative ACC for potential temperature (Theta), zonal current (UO), meridional current (VO), salinity (Sal), sea surface temperature (SST), and sea ice concentration (SIC).

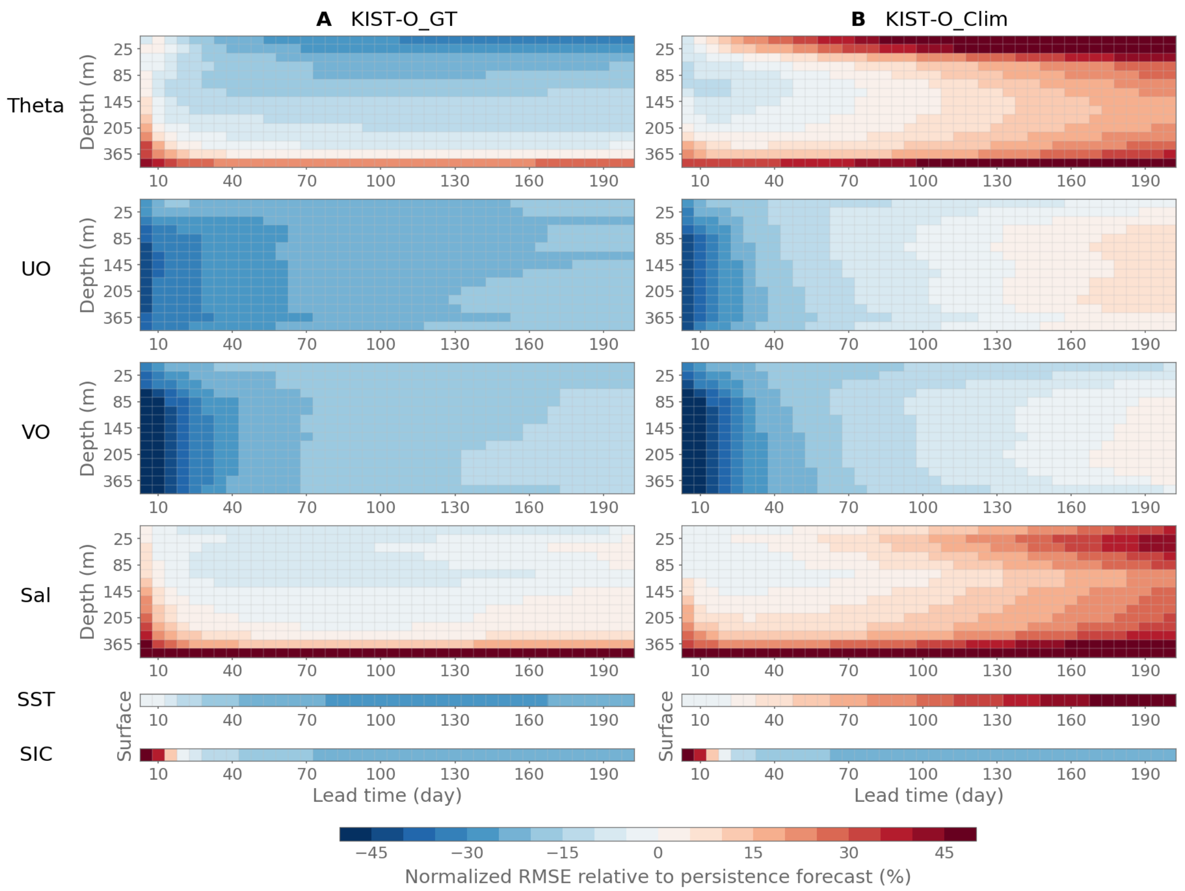

Fig. S2. Scorecard of normalized root mean square error (RMSE, %) for each KIST-Ocean inference setup relative to the persistence forecast, computed over 2014–2023. These scores are expressed as the percentage difference between the RMSE of each KIST-Ocean inference setup and that of the persistence forecast. The left column displays the normalized RMSE for KIST-O_GT, whereas the right column shows that for KIST-O_Clim. From top to bottom, each row presents the normalized RMSE for potential temperature (*Theta*), zonal current (*UO*), meridional current (*VO*), salinity (*Sal*), *SST*, and *SIC*, respectively.

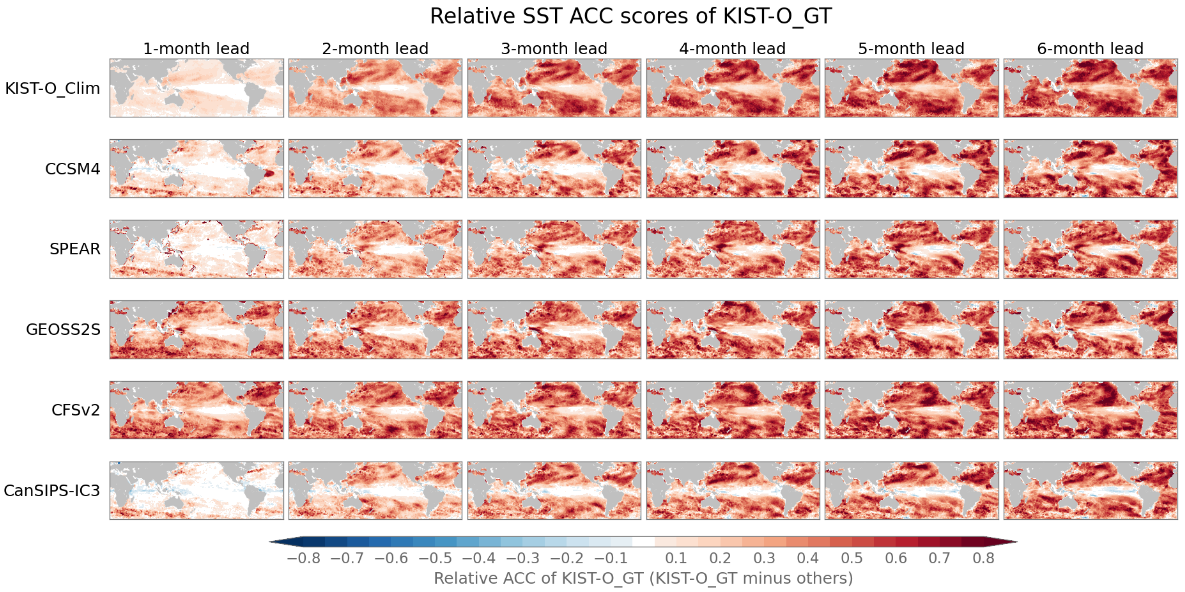

Fig. S3. Horizontal distributions of the relative anomaly correlation coefficient (ACC) for monthly *SST*, defined as the ACC of KIST-O_GT minus that of each comparison model, over the period 2015–2022. Each row (from top to bottom) represents the relative ACC of KIST-O_GT with respect to KIST-O_Clim, COLA-RSMAS-CCSM4, GFDL-SPEAR, NASA-GEOSS2S, NCEP-CFSv2, and CanSIPS-IC3, respectively. From left to right, each column represents simulations from a one-month to six-month lead time. Positive values indicate regions where KIST-O_GT outperforms the corresponding model in terms of ACC. Maps were generated using the Basemap Toolkit (v1.2.0).

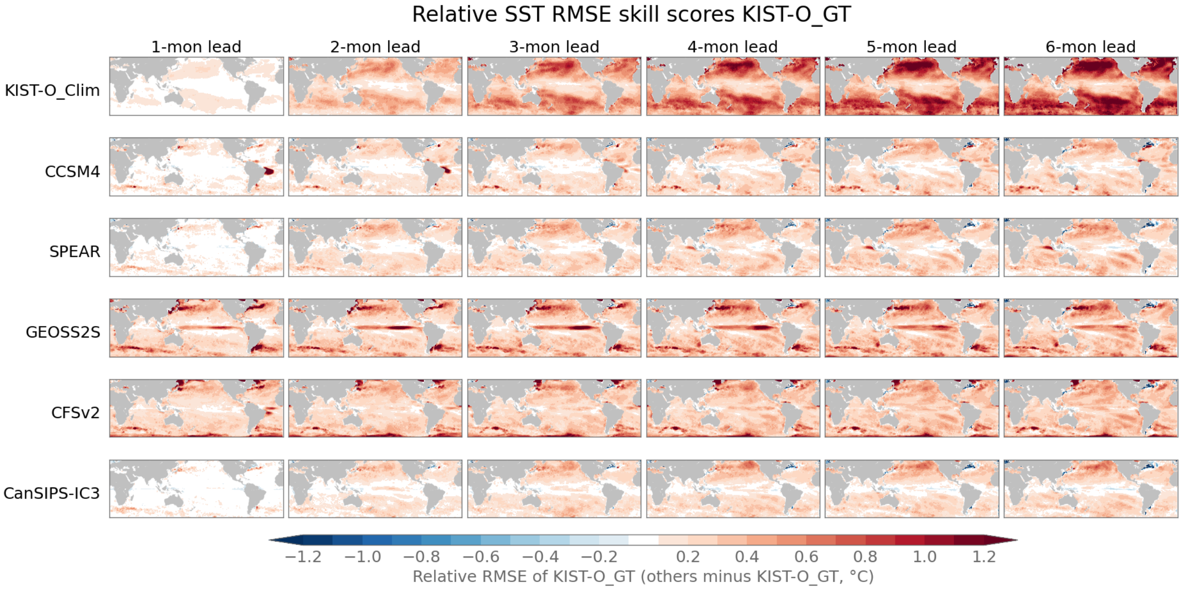

Fig. S4. Horizontal distributions of the relative root mean square error (RMSE) for monthly *SST*, defined as the RMSE of each comparison model minus that of KIST-O_GT, over the period 2015–2022. Each row (from top to bottom) represents the relative RMSE (unit: °C) of KIST-O_Clim, COLA-RSMAS-CCSM4, GFDL-SPEAR, NASA-GEOSS2S, NCEP-CFSv2, and CanSIPS-IC3, respectively. From left to right, each column represents simulations from a one- to 6-month lead. Positive values indicate regions where KIST-O_GT exhibits lower RMSE than the corresponding model. Maps were generated with the Basemap Toolkit (v1.2.0).

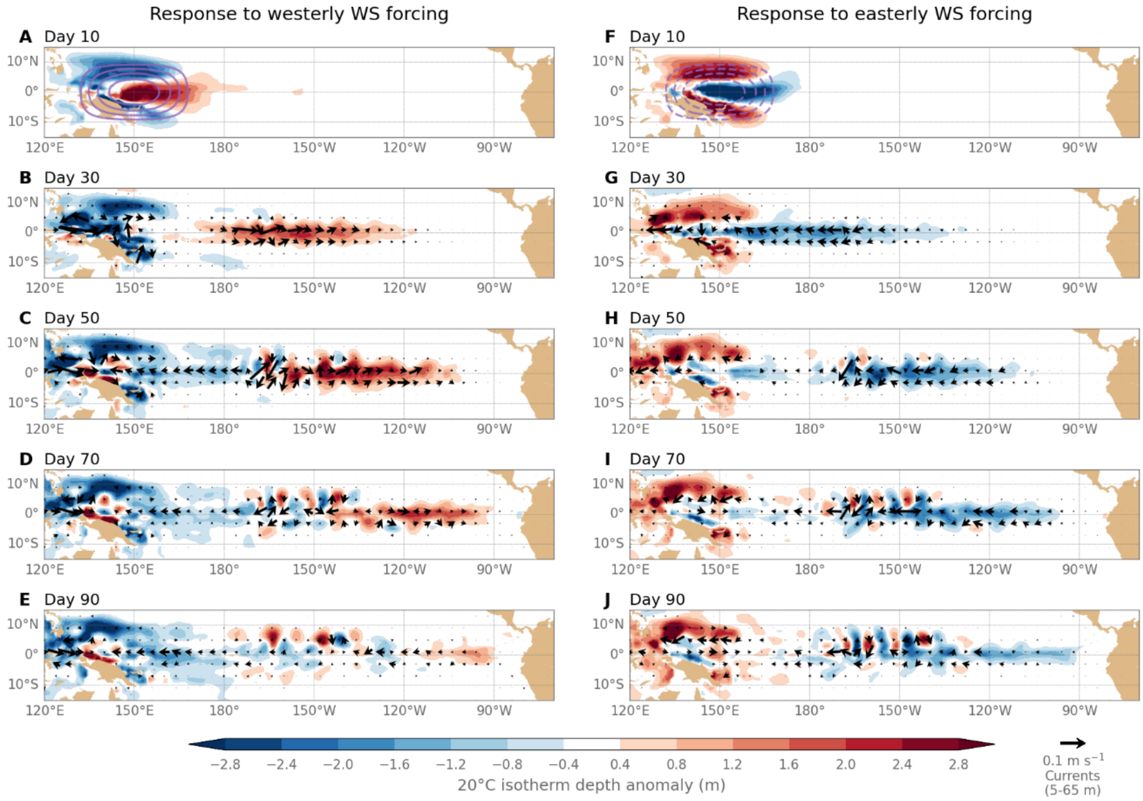

Fig. S5. Wave responses in the tropical Pacific to zonal wind stress forcing, as simulated by KIST-Ocean. The left column shows responses to westerly wind stress, while the right column presents responses to easterly wind stress. From top to bottom, each panel corresponds to lead times of 10, 30, 50, 70, and 90 d. Shading denotes the 20 °C isotherm depth anomaly (m); purple contours represent normalized zonal wind stress forcing (unitless); and black arrows denote depth-averaged (5–65 m) horizontal currents (m s^{-1}). The initial ocean state corresponds to 12–16 December 2013. For each experiment, a Gaussian-distributed forcing was nudged into the initial condition over 130°–170°E and 10°S–10°N (see "Idealized wind stress forcing nudging experiments" for details). Maps were generated using the Basemap Toolkit (v1.2.0).

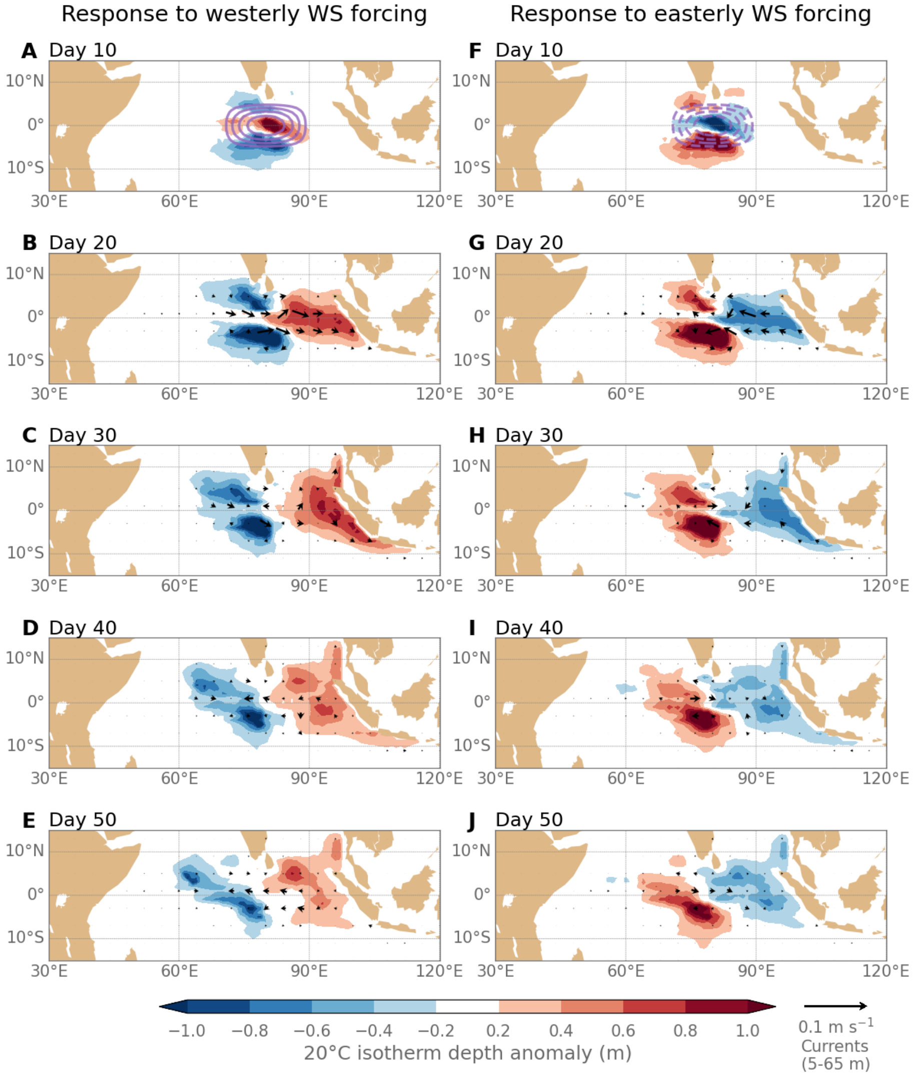

Fig. S6. Wave responses in the Indian Ocean to zonal wind stress forcing, as simulated by KIST-Ocean. The left column shows responses to westerly wind stress, while the right column presents responses to easterly wind stress. From top to bottom, each panel corresponds to lead times of 10, 20, 30, 40, and 50 d. Shading denotes the 20 °C isotherm depth anomaly (m); purple contours represent normalized zonal wind stress forcing (unitless); and black arrows denote depth-averaged (5–65 m) horizontal currents (m s^{-1}). The initial ocean state corresponds to 12–16 December 2013. For each experiment, a Gaussian-distributed forcing was nudged into the initial condition over 70°–90°E and 5°S–5°N (see “Idealized wind stress forcing nudging experiments” for details). Maps were generated using the Basemap Toolkit (v1.2.0).

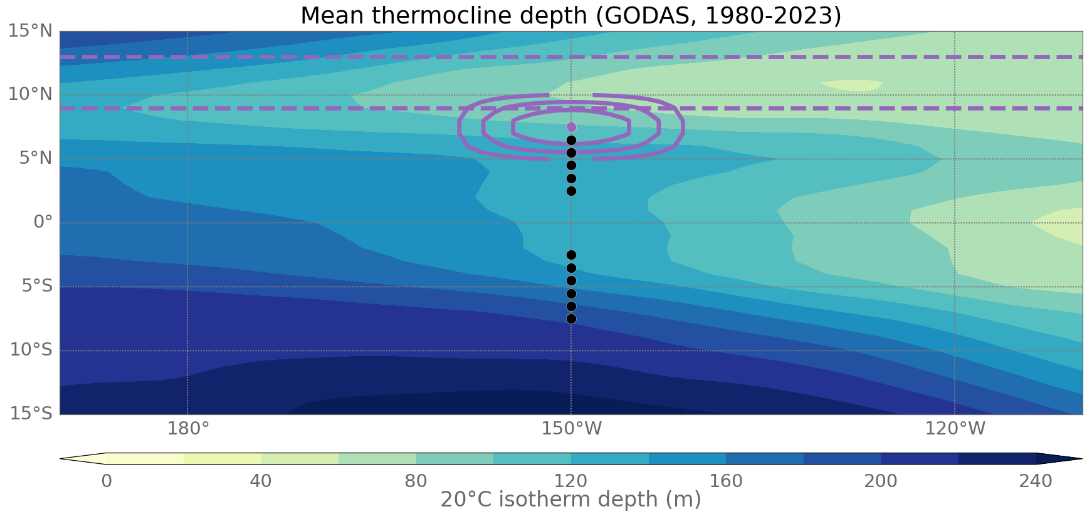

Fig. S7. Averaged thermocline depth and layout of westerly wind burst nudging experiments. Shading shows the averaged 20 °C isotherm depth over 1980–2023 (m). Dots represent the central locations of each nudging experiment (the purple dot indicates the northernmost experiment). Purple contours denote the normalized westerly wind forcing for the northernmost experiment (interval = 0.5; unitless), and the purple horizontal dashed lines indicate the Rossby wave detection zone for that experiment.

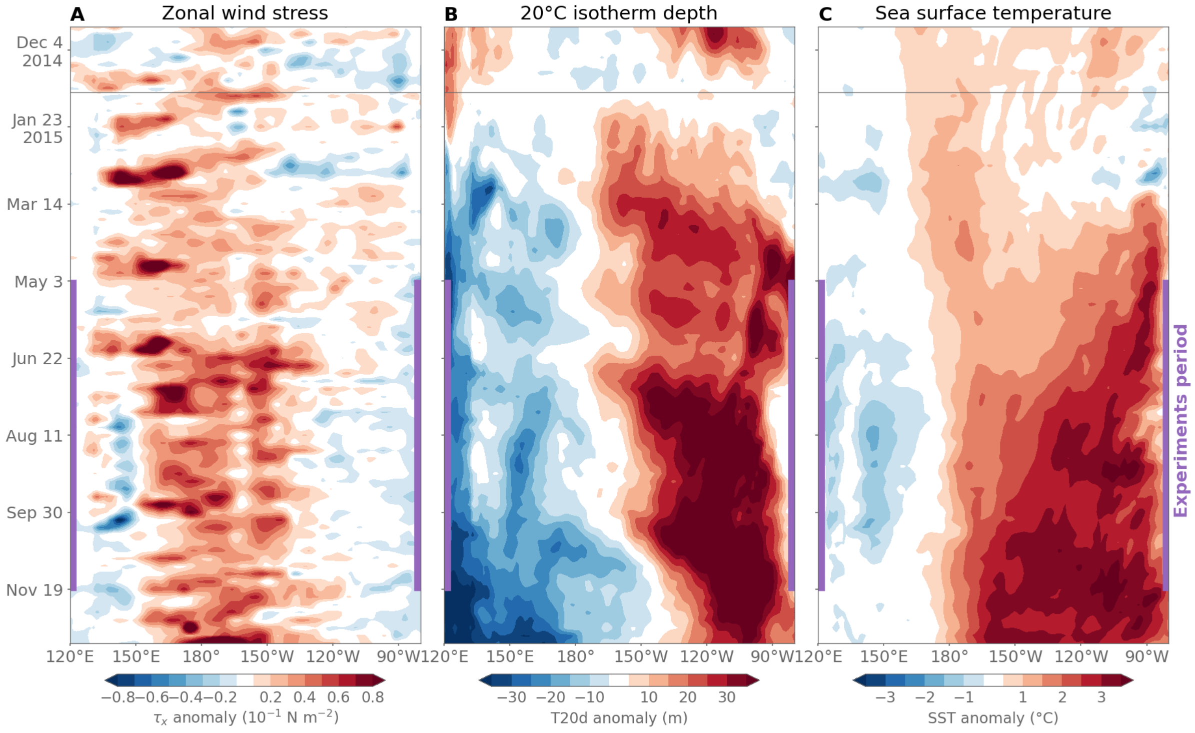

Fig. S8. Longitude-time evolution of equatorial Pacific conditions (19 November 2014–29 December 2015). Hovmöller diagrams along the equator (2°S–2°N) showing anomalies of (**A**) zonal wind stress (N m^{-2}), (**B**) 20 °C isotherm depth (m; positive = thermocline deepening), and (**C**) sea surface temperature (°C). All anomalies are computed with respect to the corresponding climatology. The purple vertical bars denote the lead time ranges of ocean simulation experiments generated with KIST-Ocean, initiated from 3 May 2015.

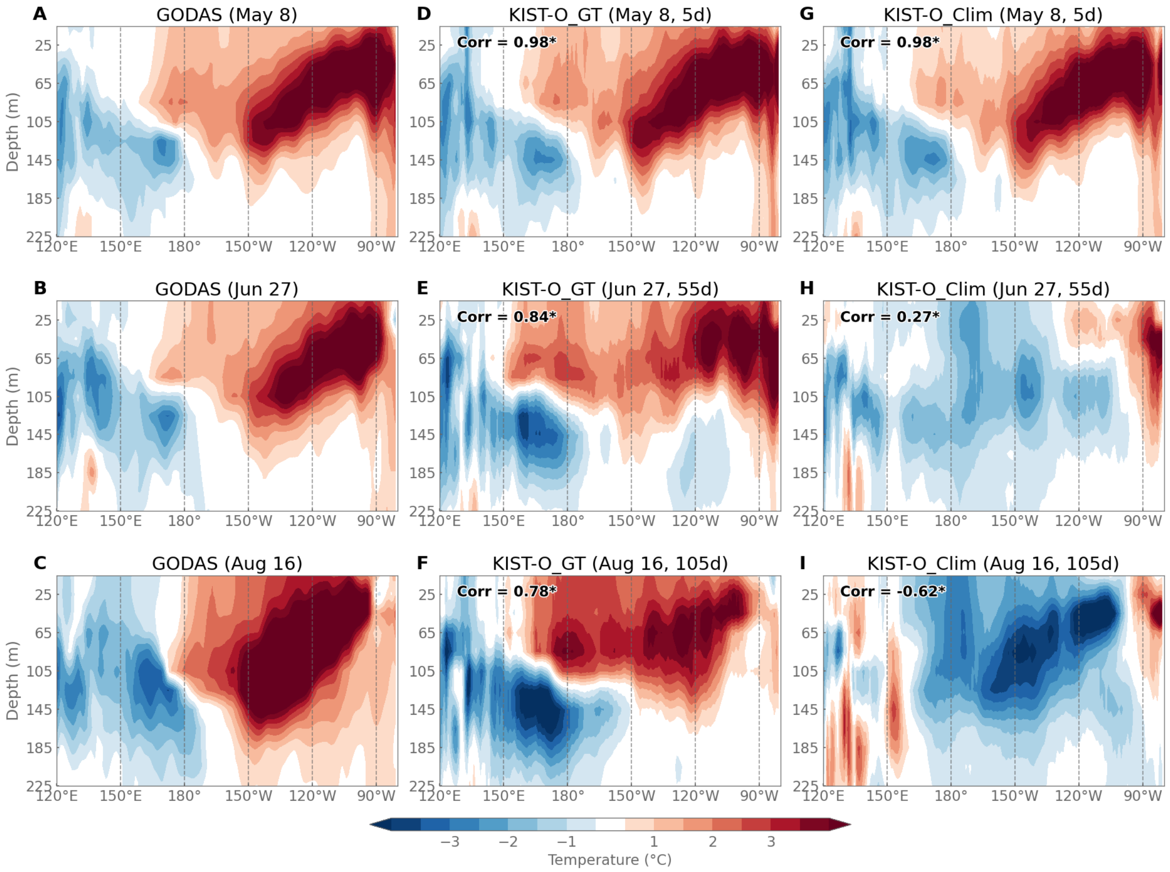

Fig. S9. Longitude–depth diagrams of pentad potential temperature anomalies (°C) along the equatorial Pacific. From left to right, the columns show the GODAS reanalysis, the KIST-O_GT simulation, and the KIST-O_Clim simulation, respectively. From top to bottom, the rows correspond to 8 May 2015, 27 June 2015, and 16 August 2015. The black numbers shown in the upper-left corner of panels D–I denote the pattern correlation coefficients with respect to the GODAS reanalysis, and asterisks indicate statistical significance at the 99% confidence level based on a Student's t-test.

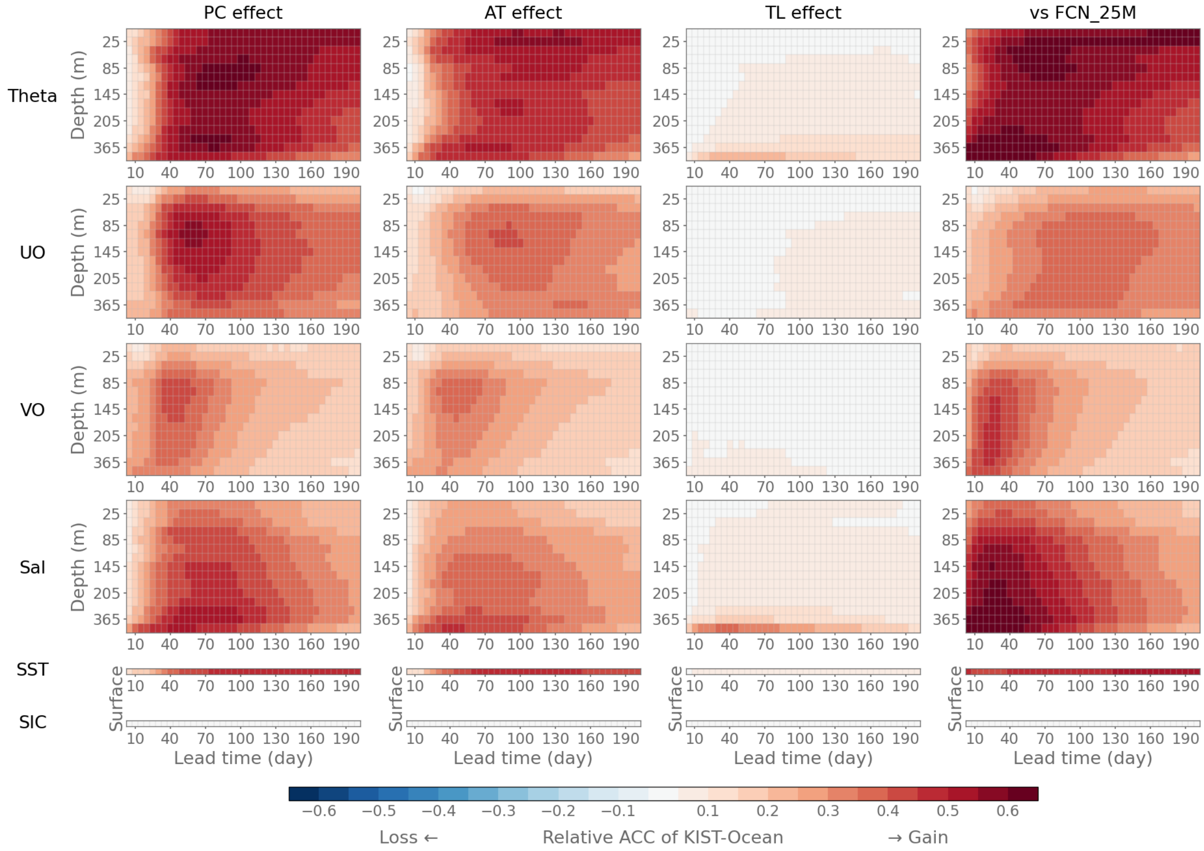

Fig. S10. Scorecard of differences in globally averaged ACC between the original version of KIST-Ocean and versions with key algorithms removed, computed over the period 2014–2023. From left to right, each column corresponds to the model without the partial convolution (PC effect), without adversarial training (AT effect), without transfer learning (TL effect), and the model based on FourCastNet with 25 million parameters (FCN_25M), respectively. From top to bottom, each row shows the ACC for potential temperature (*Theta*), zonal current (*UO*), meridional current (*VO*), salinity (*Sal*), *SST*, and *SIC*.

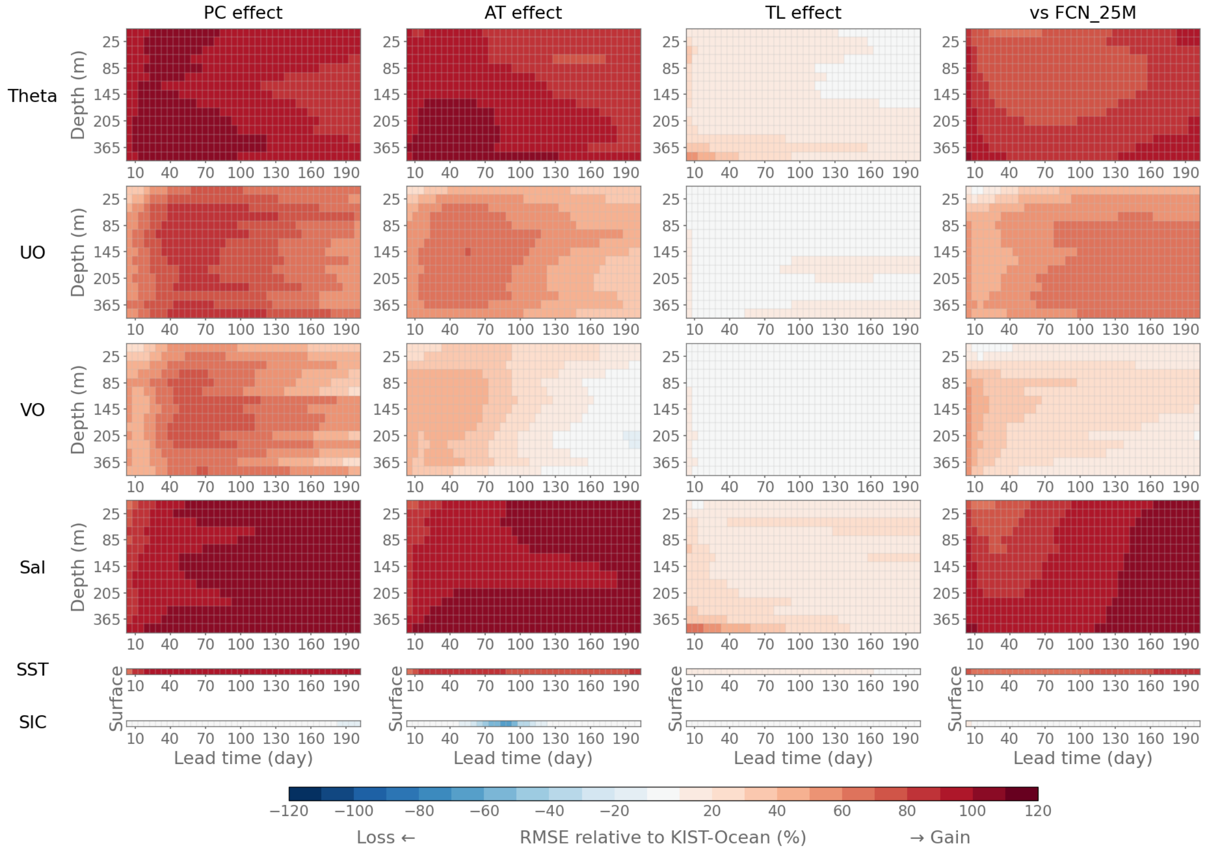

Fig. S11. Scorecard of the differences in globally averaged RMSE skills between the original version of the KIST-Ocean model and versions with key algorithms removed, computed over the period 2014–2023. From left to right, each column corresponds to the model without partial convolution (PC effect), without adversarial training (AT effect), without transfer learning (TL effect), and the model based on FourCastNet (FCN_25M), respectively. From top to bottom, each row shows the normalized RMSE for potential temperature (*Theta*), zonal current (*UO*), meridional current (*VO*), salinity (*Sal*), sea surface temperature (*SST*), and sea ice concentration (*SIC*).

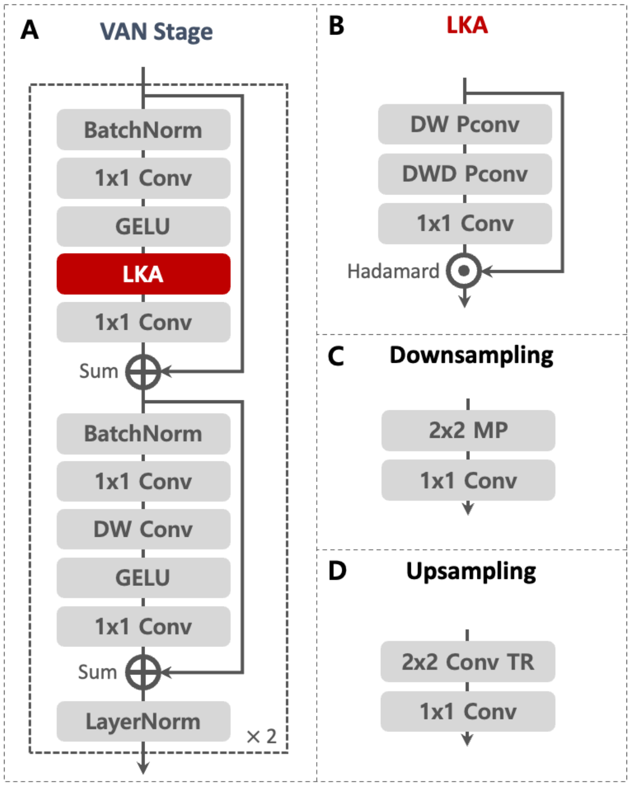

Fig. S12. Structures of the individual modules comprising the generator. (**A**) Architecture of the visual attention network (VAN) stage, where BatchNorm and LayerNorm denote batch and layer normalization, respectively, and 1×1 Conv and DW Conv denote point- and depth-wise convolutions, respectively. GELU denotes the Gaussian Error Linear Unit activation function. (**B**) Structure of the large kernel attention (LKA) module, in which DW Pconv and DWD Pconv denote depth-wise partial convolution and depth-wise dilated partial convolution, respectively. (**C**) Down-sampling module, where MP denotes max pooling that halves the x and y dimensions via a 2×2 window. (**D**) Up-sampling module, featuring Conv transposed convolution (Conv TR) to double the x and y dimensions through a 2×2 operation.

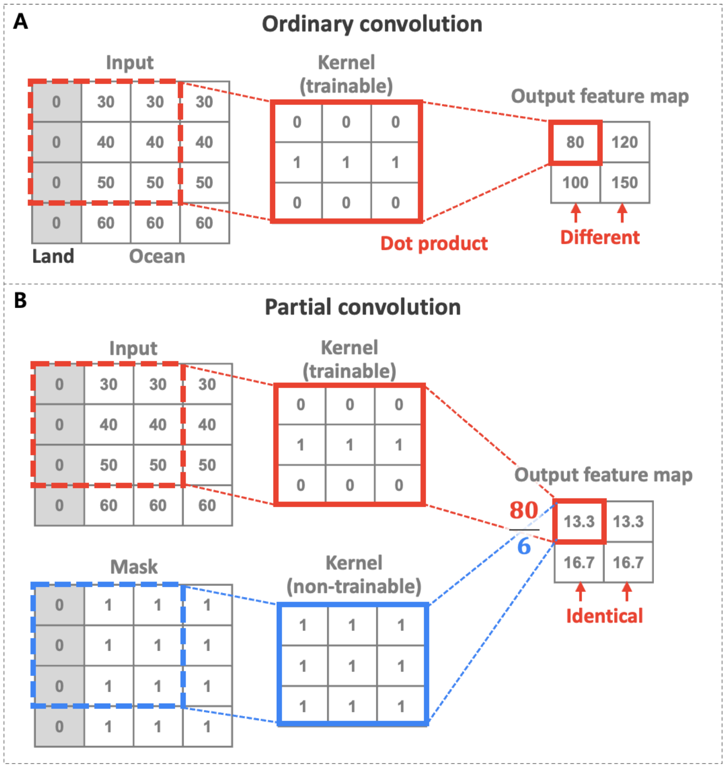

Fig. S13. Schematic illustrating the Partial convolution operation through an example. (**A**) Standard convolution. (**B**) Partial convolution. With standard convolution, the computed values at land-adjacent grid points become smaller than those at grid points farther from land. In contrast, partial convolution rescales the output to compensate for the land mask, so that values at land-adjacent grid points are not artificially reduced.

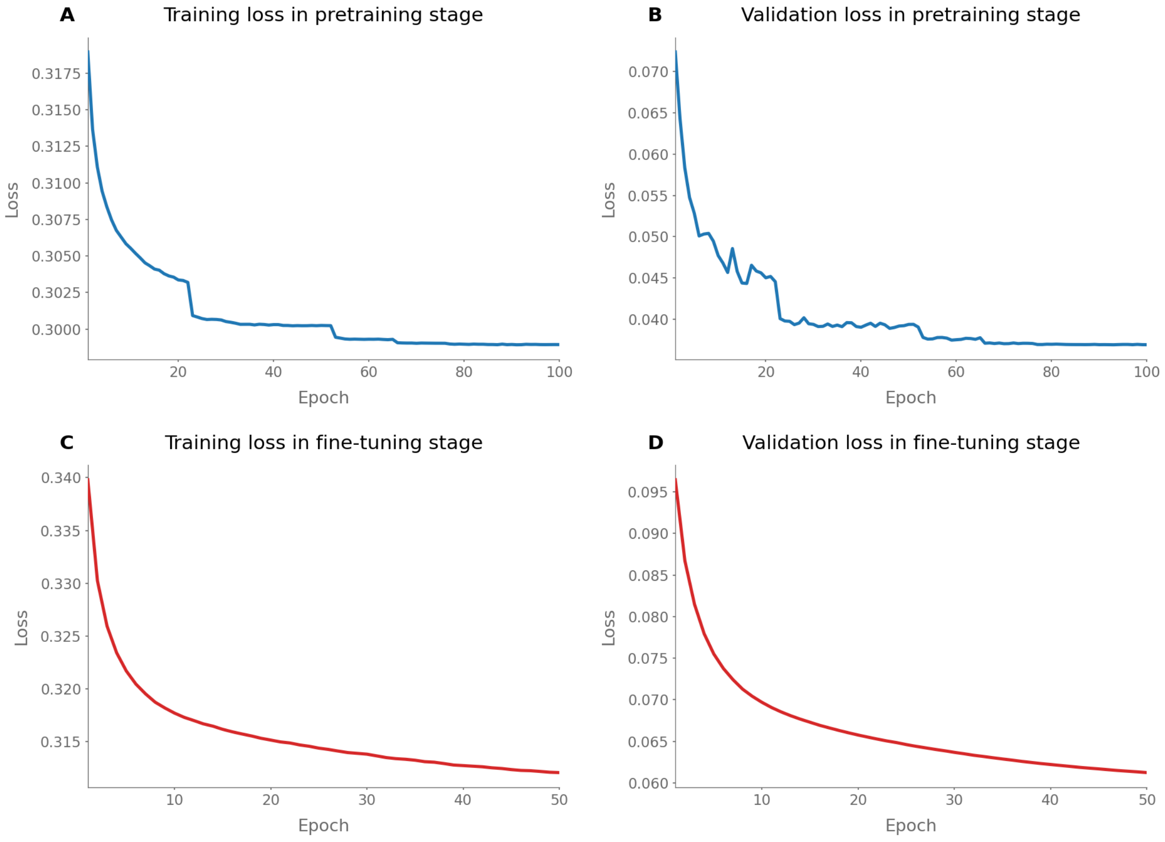

Fig. S14. Evolution of the generator loss as a function of epoch during the training of KIST-Ocean. (**A** and **B**) The training loss and validation loss during the pretraining stage, respectively. (**C** and **D**) The training loss and validation loss during the fine-tuning stage, respectively. Here, the generator loss is defined as the sum of the GAN loss and the L1 loss (see Materials and Methods).

Table S1. Ocean variables and surface boundary forcing variables used in the Korea Institute of Science and Technology's ocean model (KIST-Ocean). Please refer to the Text S1 Dataset for details.

Type	Layer	Variable	Description (unit)	Source (reanalysis)
Ocean	15	$Theta$	Potential temperature (°C)	GODAS
		UO	Zonal current (m s^{-1})	
		VO	Meridional current (m s^{-1})	
		Sal	Salinity (kg kg^{-1})	
	1	SST	Sea surface temperature (°)	OISST
		SIC	Sea ice concentration (%)	
Forcing	1	τ_x	Zonal wind stress (N m^{-2})	GODAS
		τ_y	Meridional wind stress (N m^{-2})	
		LW	Downward long-wave radiation (W m^{-2})	ERA5
		SW	Downward shortwave radiation (W m^{-2})	
		LHF	Latent heat flux (W m^{-2})	
		SHF	Sensible heat flux (W m^{-2})	
**Horizontal resolution**		1° (0°–360°, 79°S–80°N, 360×160 grid points)		
**Temporal resolution**		Pentad (5 days)		
**Depth levels (15)**		5, 25, 45, 65, 85, 105, 125, 145, 165, 185, 205, 225, 265, 365, 600 m		

Table S2. Datasets used for training and simulation skill evaluation of KIST-Ocean, including their periods and total sample counts. Note that "1301.012" and "1301.013" denote the ensemble member identification numbers from the CESM2-LE project.

	Data sources	Periods	No. of samples
**Pretraining**	CESM2-LE	1850–2014 for 1301.012<br>1850–2004 for 1301.013	23,360
	CESM2-LE	2005–2014 for 1301.013	730
**Fine-tuning**	GODAS<br>OISST<br>ERA5	1982–2013	2,336
**Testing**	GODAS<br>OISST<br>ERA5	2014–2023	730

Table S3. Latitude center of westerly wind stress forcing, forcing region, and latitude range of the Rossby wave detection zone for each nudging experiment.

Latitudinal center of forcing	Forcing region	Rossby wave detection zone
7.5°S	160°–140°W, 5°–10°S	9°–13°S
6.5°S	160°–140°W, 4°–9°S	8°–12°S
5.5°S	160°–140°W, 3°–8°S	7°–11°S
4.5°S	160°–140°W, 2°–7°S	6°–10°S
3.5°S	160°–140°W, 1°–6°S	5°–9°S
2.5°S	160°–140°W, 0°–5°S	4°–8°S
2.5°N	160°–140°W, 0°–5°N	4°–8°N
3.5°N	160°–140°W, 1°–6°N	5°–9°N
4.5°N	160°–140°W, 2°–7°N	6°–10°N
5.5°N	160°–140°W, 3°–8°N	7°–11°N
6.5°N	160°–140°W, 4°–9°N	8°–12°N
7.5°N	160°–140°W, 5°–10°N	9°–13°N

Table S4. Information on the five models from the North American Multi-Model Ensemble (NMME) project used for sea surface temperature (*SST*) simulation performance comparison with KIST-Ocean. The second column indicates the period for each model as provided by International Research Institute (IRI), and the third column shows the number of ensemble members for each model.

Model	Period	Number of Ens.
**COLA-RSMAS-CCSM4**	Jan 1982 - current	12
**CanSIPS-IC3**	Jan 1980 - current	12
**GFDL-SPEAR**	Jan 1991 - current	12
**NASA-GEOSS2S**	Feb 1982 - current	9
**NCEP-CFSv2**	Jan 1982 - current	10

REFERENCES

1. K. Bi, L. Xie, H. Zhang, X. Chen, X. Gu, Q. Tian, Accurate medium-range global weather forecasting with 3D neural networks. *Nature* **619**, 533–538 (2023).

2. R. Lam, A. Sanchez-Gonzalez, M. Willson, P. Wirnsberger, M. Fortunato, F. Alet, S. Ravuri, T. Ewalds, Z. Eaton-Rosen, W. Hu, A. Merose, S. Hoyer, G. Holland, O. Vinyals, J. Stott, A. Pritzel, S. Mohamed, P. Battaglia, Learning skillful medium-range global weather forecasting. *Science* **382**, 1416–1421 (2023).

3. L. Chen, X. Zhong, H. Li, J. Wu, B. Lu, D. Chen, S. P. Xie, L. Wu, Q. Chao, C. Lin, Z. Hu, Y. Qi, A machine learning model that outperforms conventional global subseasonal forecast models. *Nat. Commun.* **15**, 6425 (2024).

4. L. Chen, X. Zhong, F. Zhang, Y. Cheng, Y. Xu, Y. Qi, H. Li, FuXi: A cascade machine learning forecasting system for 15-day global weather forecast. *NPJ Clim. Atmos. Sci.* **6**, 190 (2023).

5. F. Ling, K. Chen, J. Wu, T. Han, J.-J. Luo, W. Ouyang, L. Bai, FengWu-W2S: A deep learning model for seamless weather-to-subseasonal forecast of global atmosphere. arXiv:2411.10191 [cs.LG] (2024).

6. J. Pathak, S. Subramanian, P. Harrington, S. Raja, A. Chattopadhyay, M. Mardani, T. Kurth, D. Hall, Z. Li, K. Azizzadenesheli, P. Hassanzadeh, K. Kashinath, A. Anandkumar, FourCastNet: A global data-driven high-resolution weather model using adaptive fourier neural operators. arXiv:2202.11214 [physics.ao-ph] (2022).

7. K. Chen, T. Han, F. Ling, J. Gong, L. Bai, X. Wang, J. J. Luo, B. Fei, W. Zhang, X. Chen, L. Ma, T. Zhang, R. Su, Y. Ci, B. Li, X. Yang, W. Ouyang, The operational medium-range deterministic weather forecasting can be extended beyond a 10-day lead time. *Commun. Earth Environ.* **6**, 518 (2025).

8. S. Rasp, S. Hoyer, A. Merose, I. Langmore, P. Battaglia, T. Russell, A. Sanchez-Gonzalez, V. Yang, R. Carver, S. Agrawal, M. Chantry, Z. Ben Bouallegue, P. Dueben, C. Bromberg, J. Sisk,

L. Barrington, A. Bell, F. Sha, WeatherBench 2: A benchmark for the next generation of data-driven global weather models. *J. Adv. Model Earth Syst.* **16**, e2023MS004019 (2024).

9. M. G. Schultz, C. Betancourt, B. Gong, F. Kleinert, M. Langguth, L. H. Leufen, A. Mozaffari, S. Stadtler, Can deep learning beat numerical weather prediction? *Phil. Trans. Royal. Soc.* **379**, 20200097 (2021).

10. Z. Ben Bouallègue, M. C. A. Clare, L. Magnusson, E. Gascón, M. Maier-Gerber, M. Janoušek, M. Rodwell, F. Pinault, J. S. Dramsch, S. T. K. Lang, B. Raoult, F. Rabier, M. Chevallier, I. Sandu, P. Dueben, M. Chantry, F. Pappenberger, The rise of data-driven weather forecasting a first statistical assessment of machine learning–based weather forecasts in an operational-like context. *Bull. Am. Meteorol. Soc.* **105**, E864–E883 (2024).

11. C. C. Liu, K. Hsu, M. S. Peng, D. S. Chen, P. L. Chang, L. F. Hsiao, C. T. Fong, J. S. Hong, C. P. Cheng, K. C. Lu, C. R. Chen, H. C. Kuo, Evaluation of five global AI models for predicting weather in Eastern Asia and Western Pacific. *NPJ Clim. Atmos. Sci.* **7**, 221 (2024).

12. M. DeMaria, J. L. Franklin, G. Chirokova, J. Radford, R. DeMaria, K. D. Musgrave, I. Ebert-Uphoff, An operations-based evaluation of tropical cyclone track and intensity forecasts from artificial intelligence weather prediction models. *Artif. Intell. Earth Syst.* **4**, e240085 (2025).

13. C. Deser, M. A. Alexander, M. S. Timlin, Understanding the persistence of sea surface temperature anomalies in midlatitudes. *J. Clim.* **16**, 57–72 (2003).

14. A. Srivastava, T. DelSole, Decadal predictability without ocean dynamics. *Proc. Natl. Acad. Sci. U.S.A.* **114**, 2177–2182 (2017).

15. Y. G. Ham, J. H. Kim, J. J. Luo, Deep learning for multi-year ENSO forecasts. *Nature* **573**, 568–572 (2019).

16. L. Zhou, R.-H. Zhang, A self-attention–based neural network for three-dimensional multivariate modeling and its skillful ENSO predictions. *Sci. Adv.* **9**, eadf2827 (2023).

17. B. Mu, B. Qin, S. Yuan, ENSO-ASC 1.0. 0: ENSO deep learning forecast model with a multivariate air–sea coupler. *Geosci. Model Dev.* **14**, 1–33 (2021).

18. B. Qin, Z. Yang, M. Mu, Y. Wei, Y. Cui, X. Fang, G. Dai, S. Yuan, The first kind of predictability problem of El Niño predictions in a multivariate coupled data-driven model. *Q. J. Roy. Meteorol. Soc.* **150**, 5452–5471 (2024).

19. R. H. Zhang, L. Zhou, C. Gao, L. Tao, A transformer-based coupled ocean-atmosphere model for ENSO studies. *Sci. Bull.* **69**, 2323–2327 (2024).

20. F. Ling, J.-J. Luo, Y. Li, T. Tang, L. Bai, W. Ouyang, T. Yamagata, Multi-task machine learning improves multi-seasonal prediction of the Indian Ocean Dipole. *Nat. Commun.* **13**, 7681 (2022).

21. W. G. Large, S. G. Yeager, The global climatology of an interannually varying air–sea flux data set. *Clim. Dyn.* **33**, 341–364 (2009).

22. M. Newman, P. D. Sardeshmukh, C. Penland, How important is air–sea coupling in ENSO and MJO evolution? *J. Clim.* **22**, 2958–2977 (2009).

23. W. J. Merryfield, W. S. Lee, G. J. Boer, V. V. Kharin, J. F. Scinocca, G. M. Flato, R. S. Ajayamohan, J. C. Fyfe, Y. Tang, S. Polavarapu, The canadian seasonal to interannual prediction system. part I: Models and initialization. *Mon. Weather Rev.* **141**, 2910–2945 (2013).

24. S. Saha, S. Moorthi, X. Wu, J. Wang, S. Nadiga, P. Tripp, D. Behringer, Y. T. Hou, H. Y. Chuang, M. Iredell, M. Ek, J. Meng, R. Yang, M. P. Mendez, H. Van Den Dool, Q. Zhang, W. Wang, M. Chen, E. Becker, The NCEP climate forecast system version 2. *J. Clim.* **27**, 2185–2208 (2014).

25. A. Molod, E. Hackert, Y. Vikhliaev, B. Zhao, D. Barahona, G. Vernieres, A. Borovikov, R. M. Kovach, J. Marshak, S. Schubert, Z. Li, Y. K. Lim, L. C. Andrews, R. Cullather, R. Koster, D. Achuthavarier, J. Carton, L. Coy, J. L. M. Friere, K. M. Longo, K. Nakada, S. Pawson, GEOS-S2S version 2: The GMAO high-resolution coupled model and assimilation system for seasonal prediction. *J. Geophys. Res. Atmos.* **125**, e2019JD031767 (2020).

26. W. Xiong, Y. Xiang, H. Wu, S. Zhou, Y. Sun, M. Ma, X. Huang, AI-GOMS: Large AI-driven global ocean modeling system. arXiv:2308.03152 [physics.ao-ph] (2023).

27. X. Wang, R. Wang, N. Hu, P. Wang, P. Huo, G. Wang, H. Wang, S. Wang, J. Zhu, J. Xu, J. Yin, S. Bao, C. Luo, Z. Zu, Y. Han, W. Zhang, K. Ren, K. Deng, J. Song, Xihe: A data-driven model for global ocean eddy-resolving forecasting. arXiv:2402.02995 [physics.ao-ph] (2024).

28. Z. Guo, P. Lyu, F. Ling, L. Bai, J.-J. Luo, N. Boers, T. Yamagata, T. Izumo, S. Cravatte, A. Capotondi, W. Ouyang, Data-driven global ocean modeling for seasonal to decadal prediction. *Sci. Adv.* **11**, eadu2488 (2025).

29. C. Wang, M. S. Pritchard, N. Brenowitz, Y. Cohen, B. Bonev, T. Kurth, D. Durran, J. Pathak, Coupled ocean-atmosphere dynamics in a machine learning Earth system model. arXiv:2406.08632 [physics.ao-ph] (2024).

30. M.-H. Guo, C.-Z. Lu, Z.-N. Liu, M.-M. Cheng, S.-M. Hu, Visual attention network. *Comput. Vis. Media* **9**, 733–752 (2023).

31. T. Li, F. Yang, Y. Song, Visual attention adversarial networks for Chinese font translation. *Electronics* **12**, 1388 (2023).

32. O. Ronneberger, P. Fischer, T. Brox, U-Net: Convolutional networks for biomedical image segmentation. Paper presented at the International Conference on Medical image computing and computer-assisted intervention, Munich, Germany (2015).

33. R. Furner, P. Haynes, D. C. Jones, D. Munday, B. Paige, E. Shuckburgh, The challenge of land in a neural network ocean model. *Environ. Data Sci.* **3**, e40 (2024).

34. I. Goodfellow, Y. Bengio, A. Courville, *Deep Learning* (MIT Press, Cambridge, 2016).

35. G. Liu, F. A. Reda, K. J. Shih, T.-C. Wang, A. Tao, B. Catanzaro, Image inpainting for irregular holes using partial convolutions. Paper presented at the European Conference on Computer Vision (ECCV), Munich, Germany (2018).

36. Y. G. Ham, Y. S. Joo, J. H. Kim, J. G. Lee, Partial-convolution-implemented generative adversarial network for global oceanic data assimilation. *Nat. Mach. Intell.* **6**, 834–843 (2024).

37. P. Isola, J.-Y. Zhu, T. Zhou, A. A. Efros, Image-to-image translation with conditional adversarial networks. Paper presented at the IEEE Conference on Computer Vision and Pattern Recognition, Honolulu, US (2017).

38. K. B. Rodgers, S.-S. Lee, N. Rosenbloom, A. Timmermann, G. Danabasoglu, C. Deser, J. Edwards, J.-E. Kim, I. R. Simpson, K. Stein, M. F. Stuecker, R. Yamaguchi, T. Bódai, E.-S. Chung, L. Huang, W. M. Kim, J.-F. Lamarque, D. L. Lombardozzi, W. R. Wieder, S. G. Yeager, Ubiquity of human-induced changes in climate variability. *Earth Syst. Dynam.* **12**, 1393–1411 (2021).

39. B. P. Kirtman, D. Min, J. M. Infanti, J. L. Kinter, D. A. Paolino, Q. Zhang, H. van den Dool, S. Saha, M. P. Mendez, E. Becker, P. Peng, P. Tripp, J. Huang, D. G. DeWitt, M. K. Tippett, A. G. Barnston, S. Li, A. Rosati, S. D. Schubert, M. Rienecker, M. Suarez, Z. E. Li, J. Marshak, Y.-K. Lim, J. Tribbia, K. Pegion, W. J. Merryfield, B. Denis, E. F. Wood, The North American multimodel ensemble: Phase-1 seasonal-to-interannual prediction; phase-2 toward developing intraseasonal prediction. *Bull. Am. Meteorol. Soc.* **95**, 585–601 (2014).

40. A. G. Barnston, M. K. Tippett, M. Ranganathan, M. L. L'Heureux, Deterministic skill of ENSO predictions from the North American multimodel ensemble. *Clim. Dyn.* **53**, 7215–7234 (2019).

41. A. Timmermann, S. Il An, J. S. Kug, F. F. Jin, W. Cai, A. Capotondi, K. Cobb, M. Lengaigne, M. J. McPhaden, M. F. Stuecker, K. Stein, A. T. Wittenberg, K. S. Yun, T. Bayr, H. C. Chen, Y. Chikamoto, B. Dewitte, D. Dommenget, P. Grothe, E. Guilyardi, Y. G. Ham, M. Hayashi, S. Ineson, D. Kang, S. Kim, W. M. Kim, J. Y. Lee, T. Li, J. J. Luo, S. McGregor, Y. Planton, S. Power, H. Rashid, H. L. Ren, A. Santoso, K. Takahashi, A. Todd, G. Wang, G. Wang, R. Xie, W. H. Yang, S. W. Yeh, J. Yoon, E. Zeller, X. Zhang, El Niño–Southern oscillation complexity. *Nature* **559**, 535–545 (2018).

42. G. Herbert, B. Bourlès, Impact of intraseasonal wind bursts on sea surface temperature variability in the far eastern tropical Atlantic Ocean during boreal spring 2005 and 2006: Focus on the mid-May 2005 event. *Ocean Sci.* **14**, 849–869 (2018).

43. P. D. Killworth, D. B. Chelton, R. A. de Szoeke, The speed of observed and theoretical long extratropical planetary waves. *J. Phys. Oceanogr.* **27**, 1946–1966 (1997).

44. Z. Ma, J. Fei, Y. Lin, X. Huang, Modulation of clouds and rainfall by tropical cyclone's cold wakes. *Geophys. Res. Lett.* **47**, e2020GL088873 (2020).

45. C. Wang, R. H. Weisberg, Ocean circulation influences on sea surface temperature in the equatorial central Pacific. *J. Geophys. Res. Oceans.* **106**, 19515–19526 (2001).

46. S. Ineson, M. A. Balmaseda, M. K. Davey, D. Decremer, N. J. Dunstone, M. Gordon, H. L. Ren, A. A. Scaife, A. Weisheimer, Predicting El Niño in 2014 and 2015. *Sci. Rep.* **8**, 10733 (2018).

47. C. C. Hong, H. H. Hsu, W. L. Tseng, M. Y. Lee, C. H. Chow, L. C. Jiang, Extratropical forcing triggered the 2015 Madden-Julian Oscillation-El Niño event. *Sci. Rep.* **7**, 46692 (2017).

48. T. Kurth, S. Subramanian, P. Harrington, J. Pathak, M. Mardani, D. Hall, A. Miele, K. Kashinath, A. Anandkumar, FourCastNet: Accelerating global high-resolution weather forecasting using adaptive fourier neural operators. Paper presented at the Platform for Advanced Scientific Computing Conference, Davos, Switzerland (2023).

49. D. W. Behringer, M. Ji, A. Leetmaa, An improved coupled model for ENSO prediction and implications for ocean initialization. Part I: The ocean data assimilation system. *Mon. Weather Rev.* **126**, 1013–1021 (1998).

50. I. Price, A. Sanchez-Gonzalez, F. Alet, T. R. Andersson, A. El-Kadi, D. Masters, T. Ewalds, J. Stott, S. Mohamed, P. Battaglia, R. Lam, M. Willson, Probabilistic weather forecasting with machine learning. *Nature* **637**, 84–90 (2025).

51. M. Long, Z. Cao, J. Wang, M. I. Jordan, Conditional adversarial domain adaptation. *Adv. Neural. Inf. Process. Syst.* **31** 1640–1650 (2018).

52. J. Yosinski, J. Clune, Y. Bengio, H. Lipson, How transferable are features in deep neural networks?. Paper presented at the Advances in Neural Information Processing Systems, Montreal, Canada (2014).

53. I. Loshchilov, F. Hutter, Decoupled weight decay regularization. Paper presented at the International Conference on Learning Representations (ICLR), New Orleans, USA (2019).

54. C. Bodnar, W. P. Bruinsma, A. Lucic, M. Stanley, A. Allen, J. Brandstetter, P. Garvan, M. Riechert, J. A. Weyn, H. Dong, J. K. Gupta, K. Thambiratnam, A. T. Archibald, C.-C. Wu, E. Heider, M. Welling, R. E. Turner, P. Perdikaris, A foundation model for the Earth system. *Nature* **641**, 1180–1187 (2025).

55. C. Kadow, D. M. Hall, U. Ulbrich, Artificial intelligence reconstructs missing climate information. *Nat. Geosci.* **13**, 408–413 (2020).

56. D. B. Chelton, M. G. Schlax, R. M. Samelson, R. A. de Szoeke, Global observations of large oceanic eddies. *Geophys. Res. Lett.* **34**, 10.1029/2007GL030812 (2007).

57. D. B. Chelton, M. G. Schlax, Global observations of oceanic Rossby waves. *Science* **272**, 234–238 (1996).

58. J. Guibas, M. Mardani, Z. Li, A. Tao, A. Anandkumar, B. Catanzaro, Adaptive Fourier neural operators: Efficient token mixers for transformers. Paper presented at the International Conference on Learning Representations (ICLR 2022), Virtual (2022).

59. N. C. Silver, W. P. Dunlap, Averaging correlation coefficients: Should Fisher's z transformation be used?. *J. Appl. Psychol.* **72**, 146–148 (1987).

60. B. Huang, C. Liu, V. Banzon, E. Freeman, G. Graham, B. Hankins, T. Smith, H.-M. Zhang, Improvements of the Daily Optimum Interpolation Sea Surface Temperature (DOISST) Version 2.1. *J. Climate* **34**, 2923–2939 (2021).

61. H. Hersbach, B. Bell, P. Berrisford, S. Hirahara, A. Horányi, J. Muñoz-Sabater, J. Nicolas, C. Peubey, R. Radu, D. Schepers, A. Simmons, C. Soci, S. Abdalla, X. Abellan, G. Balsamo, P. Bechtold, G. Biavati, J. Bidlot, M. Bonavita, G. De Chiara, P. Dahlgren, D. Dee, M. Diamantakis, R. Dragani, J. Flemming, R. Forbes, M. Fuentes, A. Geer, L. Haimberger, S. Healy, R. J. Hogan, E. Hólm, M. Janisková, S. Keeley, P. Laloyaux, P. Lopez, C. Lupu, G. Radnoti, P. de Rosnay, I. Rozum, F. Vamborg, S. Villaume, J. N. Thépaut, The ERA5 global reanalysis. *Q. J. Roy. Meteorol. Soc.* **146**, 1999–2049 (2020).
